# Smart, programmable and responsive injectable hydrogels for controlled release of cargo osteoporosis drugs

**DOI:** 10.1038/s41598-017-04956-3

**Published:** 2017-07-06

**Authors:** Konstantinos E. Papathanasiou, Petri Turhanen, Stephan I. Brückner, Eike Brunner, Konstantinos D. Demadis

**Affiliations:** 1 Crystal Engineering, Growth and Design Laboratory, Department of Chemistry, University of Crete, Heraklion, Crete, GR-71003 Greece; 2 University of Eastern Finland, School of Pharmacy, Biocenter Kuopio, P.O. Box 1627, FIN-70211 Kuopio, Finland; 3 Fachrichtung Chemie und Lebensmittelchemie, TU Dresden, 01062 Dresden, Germany

## Abstract

Easy-to-prepare drug delivery systems, based on smart, silica gels have been synthesized, characterized, and studied as hosts in the controlled release of bisphosphonates. They exhibit variable release rates and final % release, depending on the nature of bisphosphonate (side-chain length, hydro-philicity/-phobicity, water-solubility), cations present, pH and temperature. These gels are robust, injectable, re-loadable and re-usable.

## Introduction

Gel systems have found extensive applications in the medicinal/pharmaceutical field because of their ease of preparation^1^, ability for modifications^2^, and responsiveness to external chemical^3^ or physical stimuli^4^. Gels usually act as hosts for active pharmaceutical agents for a variety of pathological conditions^5^. They function as controllers of the release of pharmaceuticals that have proven to be “problematic” because they are either unsuitably insoluble to biological fluids^6^, or they are metabolized unacceptably rapidly^7^.

Among the known bone diseases (osteoporosis, osteoarthritis, multiple myeloma, Paget’s disease and several others), the most challenging is osteoporosis, which burdens millions of people compromising patients’ quality of life^8^. The recommended pharmaceutical treatment is the use of *bis*-phosphonates (BPs, a.k.a. “-dronates”)^9, 10^. Etidronic acid^11^ is the first osteoporosis treatment to enter the market (1977), while zoledronic acid^12^ is one of the treatments that followed (2007). Studies with N-containing BPs have shown that they are taken up by mature osteoclasts and inhibit farnesyl pyrophosphate synthase, an enzyme of the mevalonate pathway^13^. Their success in mitigating osteoporosis notwithstanding, these “-dronate” drugs present a number of challenges including fast excretion^14^, and numerous side-effects, such as osteonecrosis of the jaw, hypocalcemia, esophageal cancer, ocular inflammation, atrial fibrillation, etc.^15^. Nevertheless, the main drawback of BPs is their limited oral bioavailability (for example, it is ~3% for etidronic acid), which obligates physicians to increase drug intake in order to achieve the therapeutic dosage. It is, therefore, imperative to design and fabricate “smart” systems that allow controlled delivery of the active BP agent, which will depend on the patient’s needs and idiosyncrasies.

Furthermore, although all injectable BP therapies [*eg.* ibandronate (a.k.a. Bovina) and zoledronate (a.k.a. Reclast)] have been reported to alleviate the adverse effects of administration via pills/tablets, and are administered as scarcely as once a year, they are given to the patient in liquid form. The low frequency of administration of the above-mentioned BPs correlates with their very high potency as anti-resorption agents.

BP controlled release systems are scarce, however, there are some published examples. Wang *et al*. were the first to graft pamidronate onto a polymeric chain^16^. Their studies led to a new class of hydrogels containing polymeric pamidronate via crosslinking of poly(N-acryl pamidronate-*co*-N-isopropylacrylamide). A microsphere-based system of polymer-mediated, aledronate-loaded hydroxyapatite was fabricated by Huang *et al*.^17^ as a new releasing device for aledronate in bone repair applications. Chaudhari *et al*.^18^ proposed a new nanoscale targeting system which involves nanoparticles of poly(lactide-glycolide) acid, poly(ethyleneglycol) and zoledronic acid as a nanocarrier-based drug delivery system (DDS). Sundell *et al*. have prepared a poly[ethylene-g-(vinylbenzyl chloride)] film and then grafted a BP on it ref. 19. Hydrolysis of the polymer initiated the BPs release. Johnston *et al*. characterized and evaluated refillable polyurethane reservoirs with regard to release of etidronic acid *in vitro* into a receptor phase^20^. Kim *et al*. described bioabsorbable calcium phosphate microspheres that can incorporate alendronate through an *in situ* loading process and can control the alendronate release rate^21^.

It is now well-established that silica is not (cyto)toxic and has excellent biodegradation properties^22–26^. Silica has found several uses in diverse scientific and technical application fields, from food additive, to drug excipient. Furthermore, silicic acid (the dissolution product of amorphous silica) is also non-toxic. Moreover, it has been suggested as beneficial to bones^27^. The majority of studies use amorphous xerogels, mesoporous silica (such as MCM-41 or SBA-15) or fumed silica nanoparticles. Specifically, mesoporous silica nanoparticles have specific characteristics appropriate for applications such as DDSs^28^. Usually harsh conditions (elevated temperature, high pressure, and strongly alkaline or acidic solutions) are required for the fabrication of silica and particular mesoporous silica nanoparticles. Prior to loading a drug molecule, a high-temperature (~600 °C) calcination step is necessary, or template extraction using concentrated acid. As a result, these procedures raise additional costs, time and complexity in fabricating DDS^29^. Balas *et al*. tried to unravel the very attractive application of siliceous ordered mesoporous materials combined with BPs. Two types of hexagonal ordered mesoporous materials, MCM-41 and SBA-15, were used as matrices for alendronate adsorption and release^30^.

There is also widespread confusion regarding the safe use of mesoporous silica nanoparticles, especially because the interactions at the “nano-bio” interface are unknown. Therefore, it is of paramount importance to design, synthesize and fabricate DDSs for BP controlled release under mild conditions that fit an acceptable biocompatibility and toxicity profile.

In this paper, we report a detailed study on an easy-to-prepare silica hydrogel-type DDS that can host and incorporate a wide variety of BPs, and subsequently release these in a controlled manner. These hydrogels are sufficiently fluid to be injectable (see Fig. S-1, Supplementary Information, SI). Several factors have been found to influence the controlled release of the active BP, such as cations present in the gel, active groups on the BP backbone, gel density, and temperature. These systems are intended for potential biomedical applications.

## Results and Discussion

**Figure 1:**
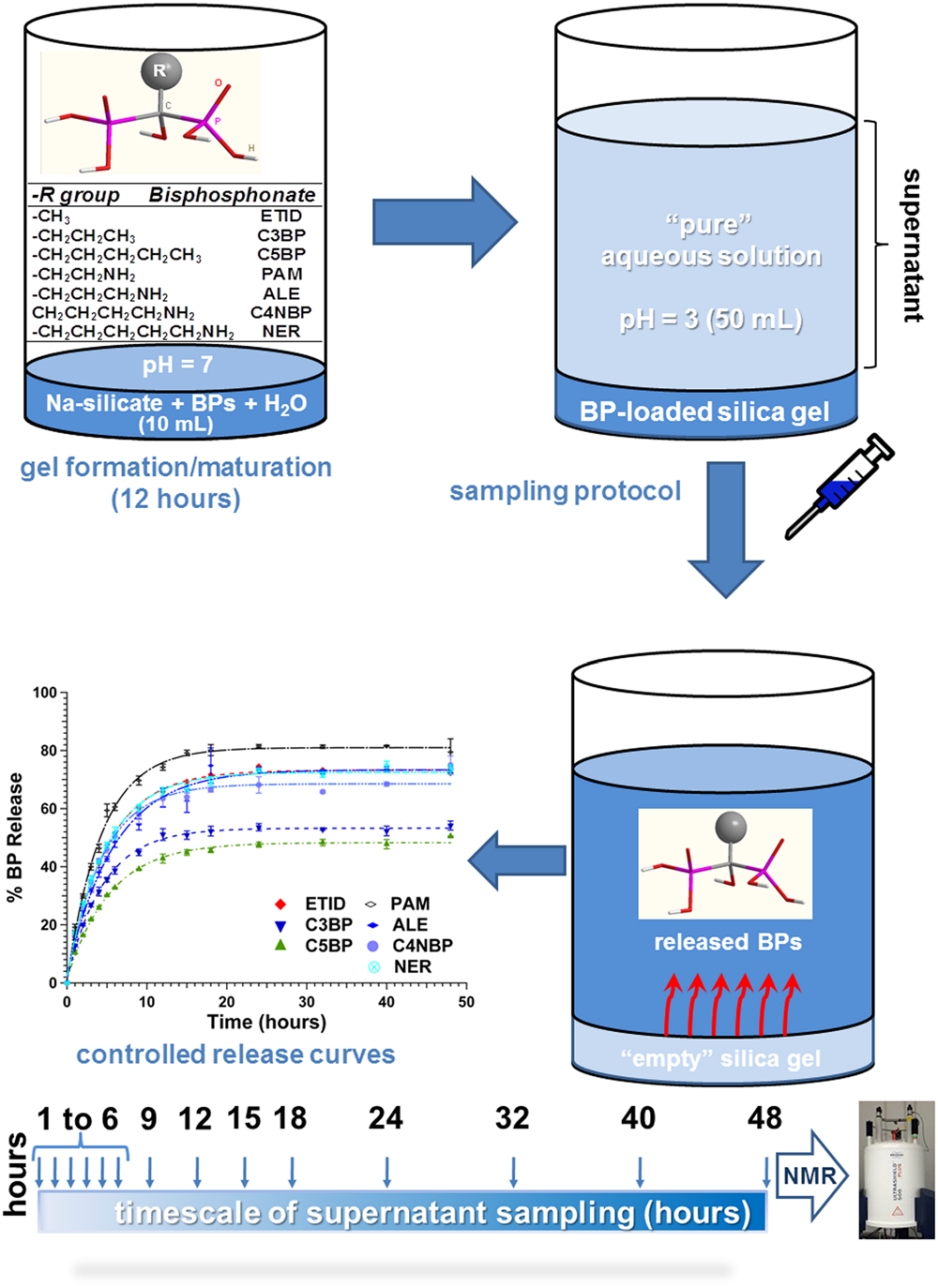
Schematic outline of gel preparation, loading, controlled release of BPs, sampling, identification and output.

For the present study seven BPs (SI, Table S-1) were used for the fabrication of BP-loaded gel DDSs. The general protocols for gel preparation, controlled release and sampling are shown schematically in Fig. 1.

All studied BPs possess the same substituent environment around the central carbon atom (two phosphonate and one hydroxyl moieties), while the R side chain is variable. Hence, the selection criteria were based on features of R such as polarity, hydrophobicity-philicity, and end-groups. Specifically, three of the BPs possess a systematically elongated hydrophobic alkyl chain (**ETID**, **C3BP**, and **C5BP**), and four display a systematically elongated aminoalkyl chain (**PAM**, **ALE**, **C4NBP** and **NER**). The importance of the BP side chain on drug effectiveness has been highlighted for commercial BPs^31^. Representative gels (“empty” *ie*. without BP, loaded with BP and finally after release) have been studied by SEM-EDS (after supercritical drying, see Figs S-2 to S-6, SI) and, as expected, they display amorphous features (by powder XRD). The presence of BP was confirmed by EDS and NMR (see below).

**Figure 2:**
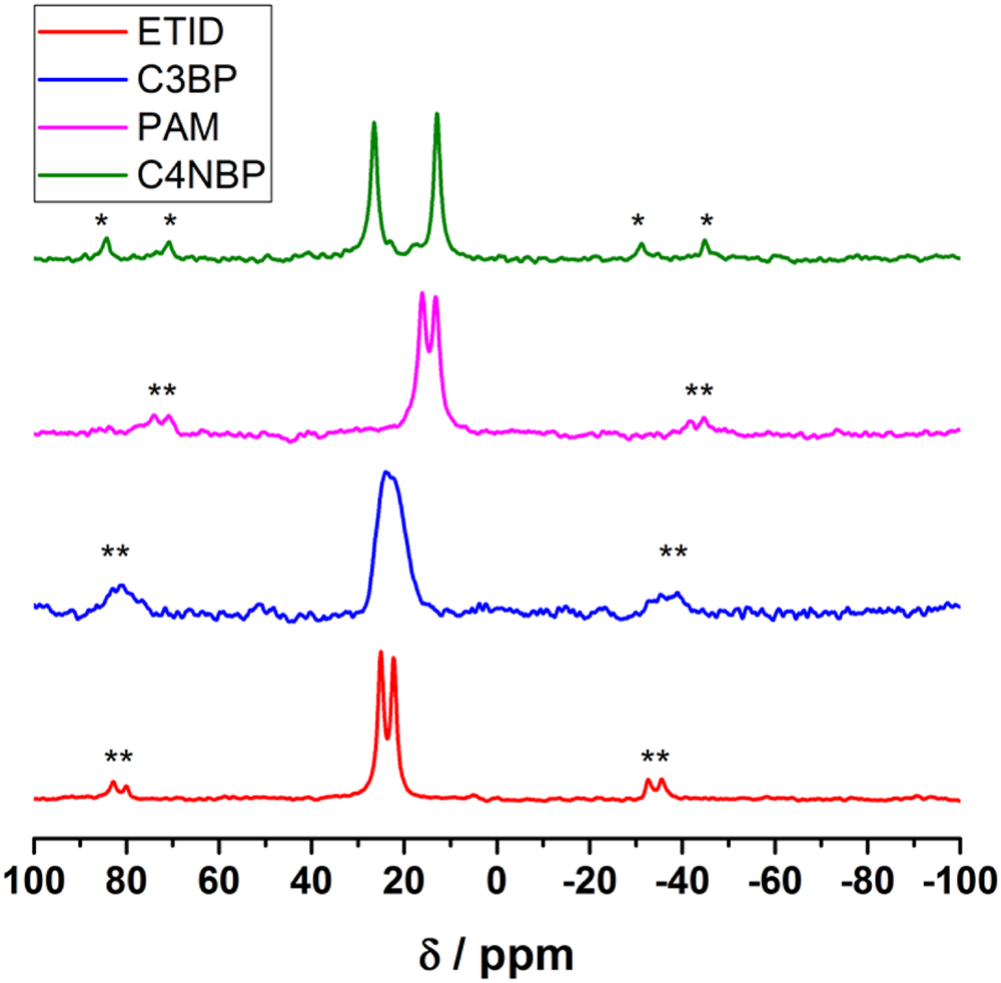
Directly excited ^31^P MAS NMR spectra of selected pure BPs before incorporation in the silica gel.

Solid state NMR (ssNMR) was used to probe the presence of the BPs in the prepared silica gels by observing the ^31^P spins. The spectra of the selected pure educts indicate different crystallographic positions of the P-containing functional groups by their isotropic chemical shift (Fig. 2). This is not uncommon, as evidenced by the crystal structures of several BPs and their Na salts^32^. Each chemically and magnetically inequivalent ^31^P contributes to a signal in the shown direct excitation MAS spectra (see Fig. 2, and data in Table 1).

**Table 1:** Isotropic chemical shifts from direct excitation ssNMR measurements for the BP educts and the species within the silica gel DDS.

Bisphosphonate	Educt δ/ppm	Gel DDS δ/ppm
ETID	22.3; 25.1	19.7
C3BP	24.8^*^; 22.2^*^	19.2
PAM	13.2; 16.1	17.9, 20.0
C4NBP	12.9; 26.5	19.0

^*^Data fitted with dmfit program “dm2011vs/rel/release #20111221”^41^.

In contrast to the dry educts, the ^31^P direct excitation MAS spectra of the BPs exhibit less lines after incorporation into the silica gels (Fig. S-37, SI, and data in Table 1). This might be due to the aqueous environment in the wet gels. Two separate peaks could only be observed for **PAM**, likely caused by the fast recrystallization of **PAM** within the silica gel in the rotor. This observation is supported by ^1^H-^31^P CP MAS measurements. In CP MAS experiments the magnetization transfer efficiency from protons to hetero nuclei is inefficient for non-rigid systems. In case of the BP-loaded silica gels, signals were exclusively found for the **PAM** sample (Fig. S-38, SI).

**Figure 3:**
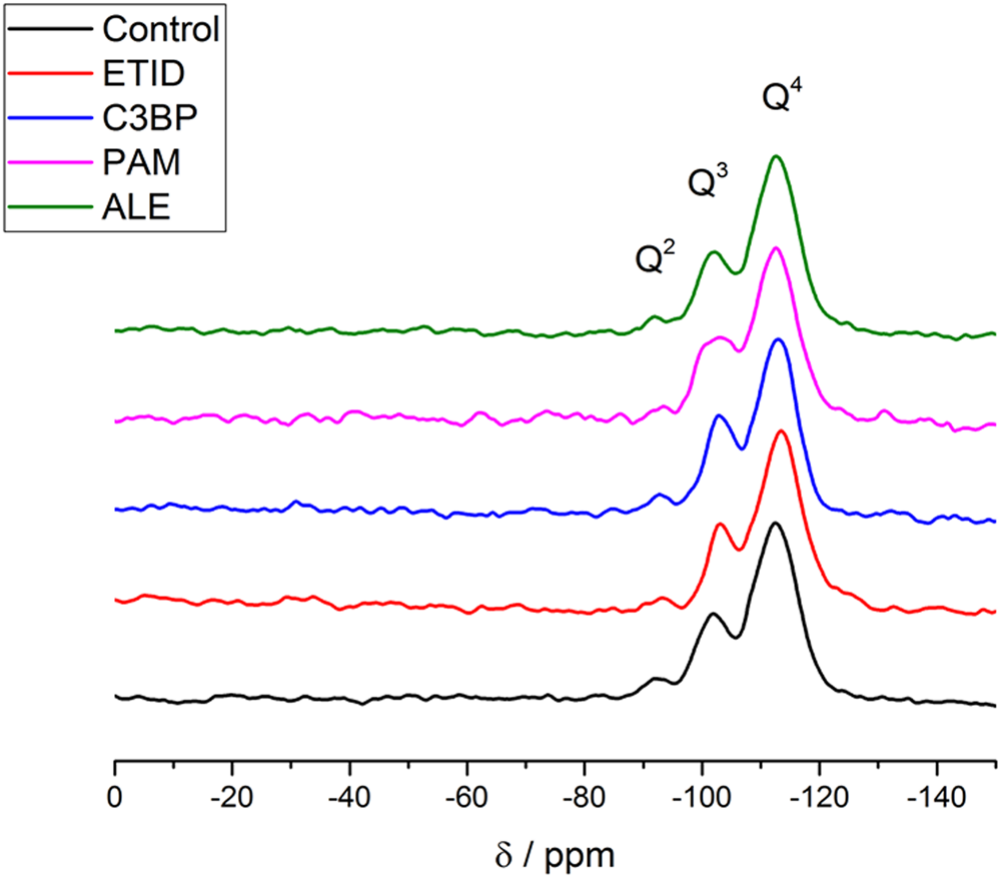
^29^Si MAS NMR (direct excitation) spectra of the various gels. No significant differences occur for the Q^n^ groups among the different gels (chemical shifts δ(Q^2^) = −91.9 ppm; δ(Q^3^) = −101.9 ppm and δ(Q^4^) = −112.6 ppm).

Furthermore, the direct excitation spectra of ^29^Si are very similar for all BP-loaded DDS gels. Specifically, there are no significant differences of the peaks caused by Q^2^, Q^3^ and Q^4^ groups occurring within the different gel samples (see Fig. 3 for selected results). Therefore, the presence of BPs seems to have no impact on the overall bulk structure of the prepared BP-loaded silica hydrogels.

Additional hydrogel characterization involved their rheological behavior. For this, three representative hydrogels were selected, “empty” (no BP drug present), ETID-loaded, and PAM-loaded. All three samples exhibited time-independent moduli during the course of time sweep measurements over 2000 s. Figure S-7 in the SI compares the subsequent (for each sample) linear viscoelastic spectra for the three samples. First, we note that the three hydrogels are very similar, exhibiting the same frequency dependence. The frequency-independent storage moduli (G’), much lower and frequency dependent (with a minimum) loss moduli (G”) and large values of G’ (order of 10^4^ Pa) indicate that the gels are strong. Typically, many colloidal gels can have much lower moduli (100 or 1000 Pa), and gels with moduli above the kPa range are strong. Secondly, we do observe quantitative differences: the “empty” hydrogel is the strongest. The ETID-loaded gel is slightly softer (note the logarithmic scale), whereas the PAM-loaded gel is clearly softer, with G’ being reduced by a factor of 2.5.

The fact that “empty” is the strongest gel is corroborated by its lower yield strain (i.e., the fractional deformation needed to break it mechanically). It is about 5%. On the other hand, the softest PAM-loaded gel is more deformable at break, having a yield strain of about 10%, whereas the intermediate ETID-loaded gel has a yield strain of 8%.

Once BP-loaded hydrogels form they do not absorb additional water. Tests (see Figure S-34 in the SI) indicate weight differences from ~0.5% to ~3.2% in hydrogels exposed to excess water. Interestingly, dried gels do not swell. When exposed to water they do not revert to hydrogels, hence the process from hydrogel to dry powder is irreversible. They also exhibited variable water re-absorption behavior (see Figure S-35 in the SI). For example an “empty” dried gel re-absorbed only ~1.3% water. However, a dried ETID-loaded gel lost ~34.6% of its weight, whereas a dried PAM-gel gained ~12.2% of its weight.

**Figure 4:**
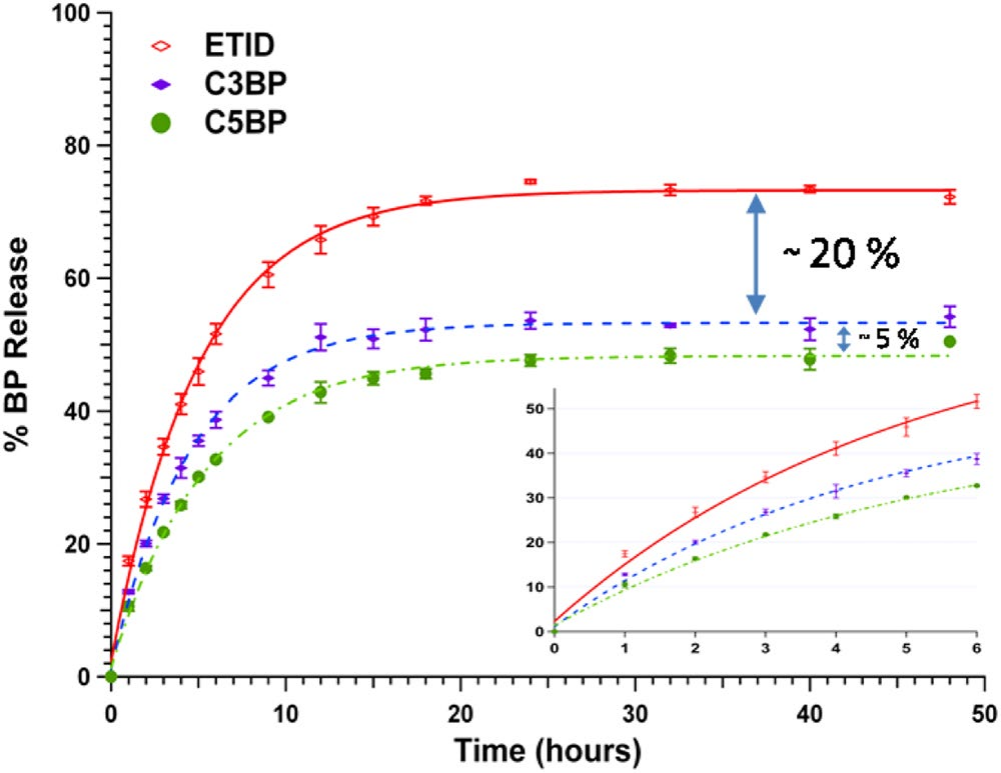
Long-term controlled release (48 hours) of BPs with aliphatic, hydrophobic side chain (**ETID**, **C3BP**, and **C5BP**).

BP-loaded gels were subjected to 48-hour controlled release experiments (each repeated at least 4 times, with excellent reproducibility, ±3% error). Release quantification was made based on ^1^H NMR signals. Figure 4 shows results on controlled release of the family of BPs with hydrophobic side-chains (**ETID**, **C3BP**, and **C5BP**).

A clear differentiation of the controlled release profiles is evident. **ETID** (with the shortest methyl side-chain) exhibits the fastest release, followed by **C3BP** (with *n*-propyl side-chain). **C5BP** (with the longest *n*-pentyl side-chain) demonstrates the slowest release rate. The final % BP released in solution after 48 hours (plateau value) follows the same ranking.

**Figure 5:**
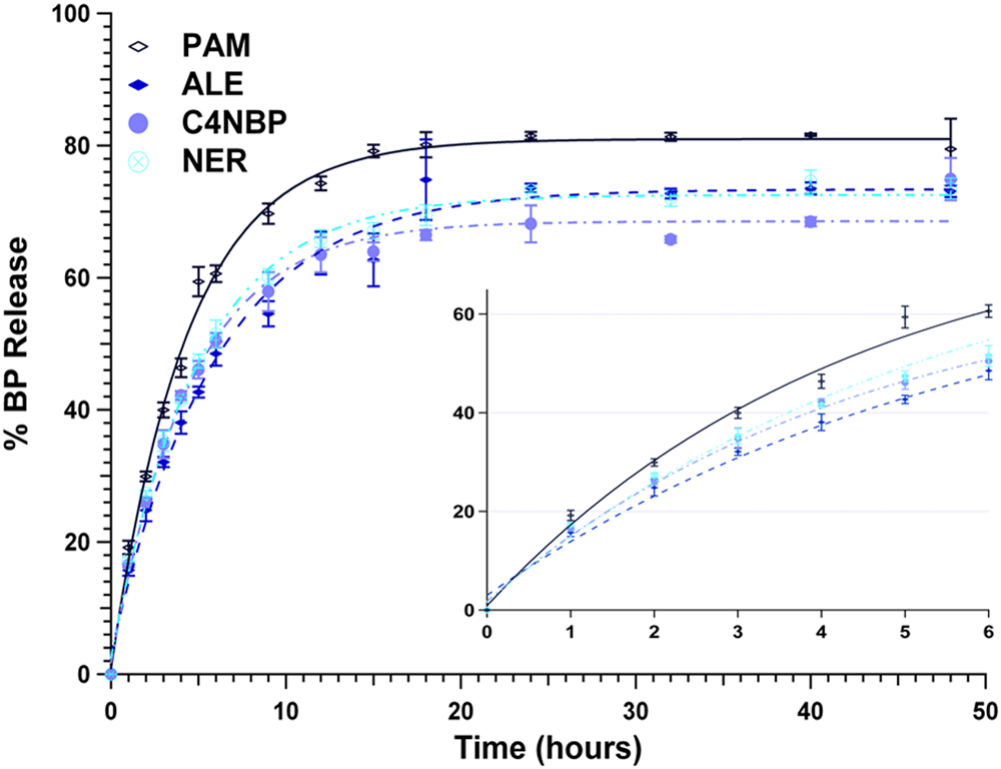
Long-term controlled release (48 hours) of BPs with hydrophilic, amine-containing side chain (**PAM**, **ALE**, **C4NBP**, and **NER**).

Figure 5 shows results on controlled release of the family of BPs with hydrophilic, amine-containing side-chains (**PAM**, **ALE**, **C4NBP**, and **NER**). The results are quite intriguing, when compared to those for non-polar side chain BPs (Fig. 4), as they reveal that the presence of the amine group on the side chain profoundly enhances release rates and the final % plateau value. **PAM**, with an ethylamine side chain, exhibits the fastest release and final % release (~80%) of all. Side chain elongation in amino-BPs decelerates release and lowers the final % plateau value (*eg*. for **C4NBP** it is 70%). Nevertheless, side-chain length increase beyond 3 atoms (two C’s, one N) does not induce systematic release reduction, as the results are nearly indistinguishable, thus pointing to a strong effect from the amine end-functionality surpassing that of the chain lengthening.

**Figure 6:**
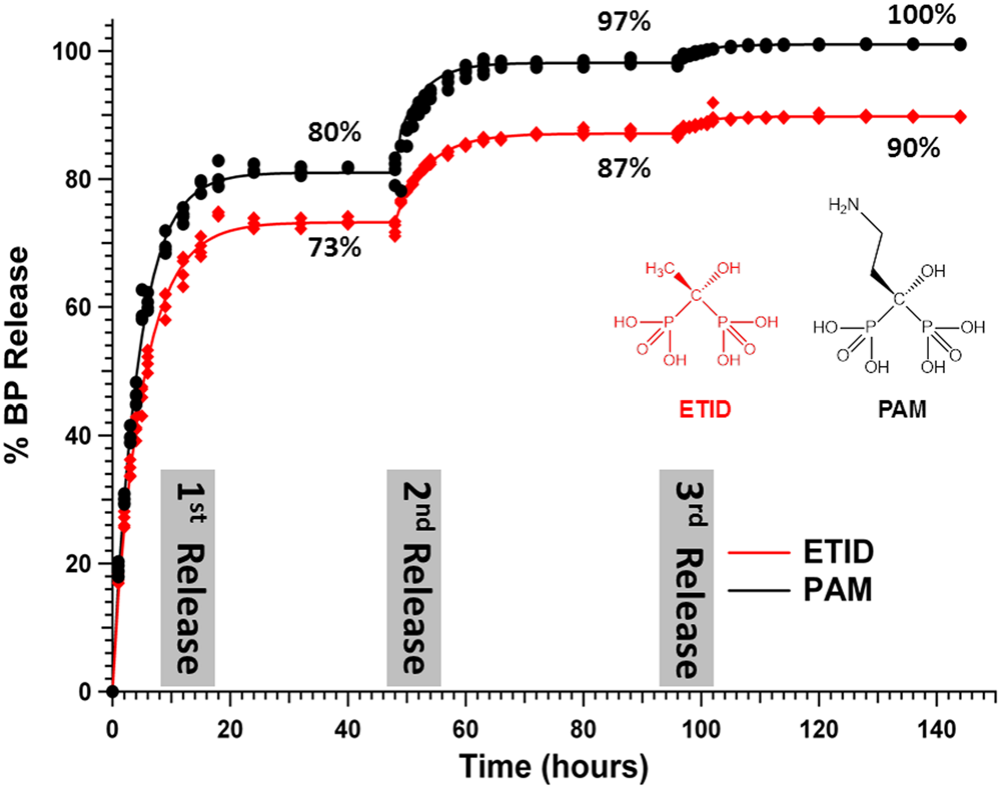
Step-wise, sustained controlled release of **ETID** and **PAM** for 144 hours.

The release results presented in Figs 4 and 5 clearly demonstrate some important trends. Both for amino-BPs and non-polar side-chain BPs, their rates and final % release correlate with their aqueous solubility trends (see Figs S-22 and S-23)^33, 34^. Interestingly, none of the BP-loaded hydrogels reaches quantitative release (*eg*. **ETID** reaches a ~75% plateau after ~24 hours). Hence, it is important to address whether the equilibrium reached after 24 h is final, and whether the remaining BP can be quantitatively delivered. Therefore, step-wise experiments were designed and carried out, in which the supernatant fluid was replaced with “fresh” aqueous medium after each release plateau was reached. The results for three step-wise release stages for the **ETID**- and **PAM**-loaded gels are shown in Fig. 6. They strongly support the conclusion that after equilibrium is “reset”, BPs’ release continues eventually reaching a final, essentially quantitative value. Significantly, there is no detectable BP entrapped in potentially inaccessible hydrogel pores, as indicated by the quantitative final release within 144 hours.

“Empty”, BP-free hydrogels were evaluated for their ability to be reloaded with drug. Thus, an “empty” (no BP) gel was prepared as “control”. This gel, together with a second BP-loaded gel *after* its release, were exposed to an aqueous supernatant that contained the same content of **ETID** (as in a regular “loaded” gel) in order to assess whether the **ETID** will re-enter the hydrogels. Indeed, both gels absorbed ~20% of dissolved **ETID**. Subsequently, both gels were subjected to the usual release conditions, delivering ~60% of the absorbed **ETID** (Fig. S-24, SI). Both gels exhibited the same BP re-absorption behavior, thus establishing that both hydrogels (“freshly-prepared” and “used”, after release) are robust and re-loadable. Although “re-loadability” may not seem to be necessary in drug/pharmaceutical applications, it may add additional functionality for environmental applications, *eg*. sustainable pollutant absorption from aqueous systems.

We have attempted to evaluate several hydrogel features in order to shed light on the factors controlling the BP release. It is important to note that the hydrogel content is principally water (in huge excess), in addition to other ions, such as Na^+^ (from the pH adjustment chemicals and the BP itself). A series of otherwise identical **ETID**-loaded gels were fabricated, but with variable amounts of Na^+^ ions (*ie*. [Na^+^]:[**ETID**] molar ratio ranging from 2 to 6), and was found that the Na^+^ ion concentration does not affect BP release (provided the pH is not altered), see Fig. S-25, SI.

It appears that the BP release profile (rate and final % value) can be fine-tuned by modifying a number of variables. Although the pH of the aqueous phase starts with low values (pH = 3, to mimic the human stomach), it quickly acquires its equilibrium value of ~7, regardless of the BP loading (Fig. S-26, SI). However, if the equilibrium pH is higher (*eg*. 10.5) the BP release is much slower (Fig. S-27, SI).

In order to assess the influence of nature of the alkali cation present in the hydrogel, we fabricated gels in the presence of larger ionic radius K^+^ and Rb^+^ cations (it was impossible to prepare gels with Li^+^ and Cs^+^ cations). It appears that cation replacement (Na^+^ by K^+^) decelerates the release and causes lower BP final % release (Fig. S-28, SI). Unfortunately, the Rb^+^ gel gave complicated results. It is thus unclear how precisely the alkali cations affect release. Gel release curves were generated at different temperatures revealing that the release is enhanced as T increases (Fig. S-28, SI).

In an effort to further decelerate BP release, more dense hydrogels were prepared, by changing (doubling) the sodium silicate concentration from 6.66% to 13.32%. However, the release rates and final % release plateaus were not systematic (Figure S-33). In the “dense” **ETID**-loaded gel a higher release rate and final release value were observed. In contrast, in the “dense” **PAM**-loaded gel a lower release rate and final release value were noted. This is an intriguing point that needs further systematic experimentation.

All hydrogels collapse upon water loss and eventual drying. Three representative dried gels (“empty” without BP drug present, ETID-loaded, and PAM-loaded) were used for N_2_ sorption measurements in order to obtain nitrogen sorption isotherms at 77 K and BET plots (Figures S-8 and S-9). These data revealed that the specific surface areas (SSA) for the three dried gels are: 83.4 m^2^/g for the “empty gel, 41.2 m^2^/g for the **ETID**-loaded gel and 7.4 m^2^/g for the **PAM**-loaded gel. The downward trend is consistent with the BP drug partially filling the voids within the gel, a fact that is corroborated by the observation that when BP molecular size increases (from **ETID** to **PAM**) the SSA drops from 41.2 m^2^/g to 7.4 m^2^/g. The fact that upon **ETID** loading the SSA for the “empty” gel drops from 83.4 m^2^/g to 41.2 m^2^/g is consistent with the SEM images shown in the SI. It is interesting to note that both drug-loaded **ETID** and **PAM** dried gels exhibit a “burst” release of the active drug (>90%) within a couple hours (results not shown).

Comparison of BP release (both initial rates and final “plateau” values) between the two families of BPs with non-polar alkyl and aminoalkyl side-chains is quite revealing. Presence of the amine group profoundly enhances release. This is true for similar molecular size BPs, exhibiting “short” 3-atom, and also for “long” 5-atom chain lengths (Figs S-30 and S-31, SI). Phosphonate interactions with silica surfaces have been studied in detail^30, 35^. It could be envisioned that the loaded BPs can interact with the gel internal surface through hydrogen-bonding, in which both silanol and phosphonate moieties can participate. Based on ^29^Si MAS NMR spectra the Q2 + Q3% content in various gels can range from 22% to 30% (Fig. S-32, SI). In other words, there are sufficient silanol groups to form hydrogen bonds with the oxygen-rich phosphonate groups. We have also attempted to draw useful information on silica-BP interactions from comparisons between FT-IR spectra of dried gels and “authentic” (free) BPs. However, the results are not conclusive. In general, bands due to a variety of vibrational modes of BPs are present in the spectra of dried gels. The main bands assigned to the –PO_3_H^−^ moieties (~900–1100 cm^−1^) overlap with the hydrogel’s Si-O-Si bands in the same region. Hence, several fine features of the phosphonate vibrational modes disappear due to this overlap. Furthermore, the bands assigned to the R-(CH_2_)_x_-NH_3_^+^ moiety (in the regime >3000 cm^−1^) broaden and are undetectable in the dried gel spectra. Regarding the protonation state of the BPs, it is certain (from literature reports^36–38^) that at pH 7 (the pH of the internal gel regions) each phosphonate moiety is mono-deprotonated (–PO_3_H^−^), and (for the aminoalkyl BPs) the amine moiety is protonated (R-(CH_2_)_x_-NH_3_^+^).

The release profiles of the alkyl-BPs and amino-BPs (with the latter demonstrating faster and higher release) are inversely proportional to the aqueous solubility of these two families of BPs. Amino-BPs are much less soluble in water than alkyl-BPs^33, 34^, most likely due to the extensive intermolecular H-bonding interactions of the protonated amine moiety. However, our results reveal that they are unexpectedly delivered more readily to the aqueous phase than their alkyl-BP analogs. Assuming that they are anchored to the gel internal surfaces through their bisphosphonate moiety, they leave their protonated amino (R-NH_3_^+^) moiety protruding towards the aqueous phase. Strong hydration of the -NH_3_^+^ group augments the eventual detachment of the amino-BPs and their final release from the silica surface. Hydrogen bonds between water and the -NH_3_^+^ group have been reported in the solid state^39^.

## Conclusion

In conclusion, we have reported the fabrication, characterization and BP-release properties of silica-based gels. These gels can be prepared in a cost-effective manner from cheap reagents. They possess several attractive features such as injectability, responsiveness to temperature, re-usability, and re-loadability. Furthermore, the BP drug release profiles can be fine-tuned to achieve the desired drug release, by altering several factors, such as temperature, cations present, pH and structural features of the BPs.

## Methods

### Materials

Sodium silicate pentahydrate, Na_2_SiO_3_·5H_2_O, silicic acid (<20 micron, refined, 99.9%) and potassium hydroxide was purchased from Sigma Aldrich. Rubidium hydroxide hydrate was purchased from Alfa-Aesar. **ETID** (either as solid tetrasodium salt or as acid in aqueous solution) was used as received from Solutia Inc. Deuterium oxide (99.9 atom % D) that contained 0.05 wt. % 3-(trimethylsilyl)propionic-2,2,3,3-d4 acid, sodium salt (TSP) purchased also from Sigma-Aldrich. Deionized water from an ion-exchange resin was used for all experiments and stock solution preparations.

### Instrumentation

Solid state NMR experiments were performed on a Bruker Avance 300 NMR spectrometer with a 7 mm MAS wide bore probe. The operating resonance frequency (rf) of 300.1 MHz for ^1^H, 59.6 MHz for ^29^Si and of 121.5 MHz for ^31^P measurements. 7 mm Zirconia rotors with KEL-F inserts were used. For direct excitation measurements π/2 pulses at rf fields of 34.7 kHz on ^29^Si and 65.8 kHz on 31 P were applied. The interscan delay was set to 180 s for ^31^P and 120 s for ^29^Si. During acquisition TPPM decoupling with rf field at 50.0 kHz was used. Measurements were performed at a sample spinning speed of 7 kHz. For the ^1^H-^31^P CP MAS experiments a ramped CP by using an rf field of 53.2 kHz for the spinlock on ^31^P, an 80–100% ramp on the proton channel, and a contact time of 1.5 ms were applied. The corresponding decoupling was performed using the TPPM decoupling scheme at an rf field of 50.0 kHz during acquisition. The interscan delay was set to 5 s. An AVANCE 300 (Bruker, Karlsruhe, Germany) spectrometer was used for the BP release experiments. SEM data and images collected with a JOEL JSM-6390LV electron microscope.

### Nitrogen sorption measurements

Low-pressure N_2_ sorption measurements were carried out on an Autosorb 1-MP instrument from Quantachrome equipped with multiple pressure transducers for highly accurate analyses and an oil-free vacuum system. Ultra-high purity grade N_2_ (99.999%) was used for all adsorption measurements. Prior to analysis, each sample was activated by heating at 80 °C under dynamic vacuum for 12 hours. After evacuation, the sample and cell were re-weighed to obtain the precise mass of the evacuated sample. Finally, the cell was transferred to the analysis port of the gas adsorption instrument.

### Bisphosphonate synthesis

**ALE** and **NER** were synthesized and characterized as reported elsewhere^29^. **C3BP** (disodium salt) and **C5BP** (disodium salt) were synthesized according to the procedure described by Egorov *et al*.^40^. Further BP synthesis details are given in the SI.

### Preparation of gels

Herein, the synthesis of an **ETID**-loaded gel is described, as an example. All other BP-loaded gels were prepared in the same manner. The synthesis of each BP-loaded gel was repeated four (4) times using identical shape and diameter borosilicate glass beakers. In a beaker 10 mL of DI water was added. In this a quantity (0.66 g, 3.14 mmol) of sodium metasilicate pentathydrate was dissolved, together with 0.50 g, 1.70 mmol) of tetrasodium **ETID**, while keeping the solution under stirring. The pH value of this solution was ~12.5. The pH was adjusted to 7.00 with the use 0.75 mL of concentrated HCl (37%). This particular pH value was selected because the polymerization of silicic acid has the highest rate there. Gel formation commences within 10 minutes (see video clip in the SI), however the freshly formed and “loose” gel was allowed to mature for 12 hours, after which a shapely and translucent gel formed. Gel preparation can be reproducibly repeated and can be modified by altering the amount of Na^+^ ions, replacing the alkali ion, or changing the entrapped BP. BP-containing gels for all remaining BPs were prepared in the same manner, using quantities shown in the experimental details in the SI.

### Gel rheological studies

Measurements were performed in a strain-controlled rotational rheometer (ARES 100FRTN1 from TA, USA), with stainless steel parallel plate geometry (diameter 10 mm) at 25 °C. The temperature was controlled by means of a recirculating fluids bath (water/ethylene glycol mixture). Each sample was placed between the plates with a thickness of about 1 mm. It was appropriately trimmed at the edge. To eliminate the risk of solvent (water) evaporation, the region around the samples’ edge was effectively sealed with silicon oil (viscosity 5 mPa) by means of a coaxial home-made outer aluminum ring that contained it. Linear viscoelastic measurements were performed in order to characterize the different hydrogels. The protocol involved dynamic time sweeps to ensure equilibration of the samples, strain sweeps to determine linear viscoelastic limit and frequency response to obtain the spectra.

### Controlled release of BPs from gels

On top of the solidified gel (see above), a volume of DI water (50 mL), pre-acidified to pH ~3 was carefully poured. This marked the initiation of the controlled release process (t = 0), which continued for 48 hours. For the initial 6-hour period an aliquot of 0.350 mL was withdrawn from the supernatant every hour. After the 6^th^ hour and for the next 12 hours, sampling was performed every 3 hours. Finally, after the 18^th^ hour and until the end of the release experiment (at the 48^th^ hour) sampling was performed every 8 hours. The withdrawn samples were mixed with 0.150 mL of deuterium oxide (99.9 atom % D) that contained 0.05 wt. % (4.3375 μmol) 3-(trimethylsilyl)propionic-2,2,3,3-*d*_4_ acid, sodium salt, TSP) as standard. ^1^H NMR spectra were recorded on a Bruker AVANCE 300 MHz NMR (Bruker, Karlsruhe, Germany) spectrometer at 293.2 K operating at a proton NMR frequency of 300.13 MHz. Standard solvent (D_2_O) was used as internal lock. Each ^1^H spectrum consisted of 32 scans requiring 3 min. and 39 min. acquisition time with the following parameters: Spectral width = 20.5671 ppm, pulse width (P1) = 15.000 μs, and relaxation delay (D1) = 4.000 seconds. Polynomial 4^th^-order baseline correction was performed before manual integration of all NMR spectra. Proton and carbon chemical shifts in D_2_O are reported relative to TSP. The characteristic peaks for each compound were integrated using the integration tool available from the Bruker software (TopSpin 3.2). For each compound we selected the integration value of the sharpest peak. All the integration values were cross-checked in order to ensure the best result for each compound (see spectra in the SI).

### Data Availability

The datasets generated and analyzed during the current study are available from the corresponding author on reasonable request.

## Electronic supplementary material

Formation of an ETID-loaded hydrogel.

Supplementary Information

## References

[1] Demirci, U. & Khademhosseini, A. (Eds) *Gels Handbook: Fundamentals, Properties and Applications*, World Scientific Publishing, Hackensack, NJ (2016).

[2] Weiss, R. G. & Terech, P. (Eds) *Molecular Gels: Materials With Self-Assembled Fibrillar Networks*, Springer, Heidelberg (2006).

[3] Ahn S-K, Kasi RM, Kim S-C, Sharma N, Zhou Y (2008). Stimuli-responsive polymer gels. Soft Matter.

[4] Li X, Gao Y, Serpe MJ (2016). Stimuli-responsive assemblies for sensing applications. Gels.

[5] Rao KM, Rao KSVK, Ha C-S (2016). Stimuli responsive poly(vinyl caprolactam) gels for biomedical applications. Gels.

[6] Hoare TR, Kohane DS (2008). Hydrogels in drug delivery: Progress and challenges. Polymer.

[7] Liechty WB, Kryscio DR, Slaughter BV, Peppas NA (2010). Polymers for drug delivery systems. Annu. Rev. Chem. Biomol. Eng..

[8] Madureira MM, Ciconelli RM, Pereira RMR (2012). Quality of life measurements in patients with osteoporosis and fractures. Clinics.

[9] Poole KE (2012). Bisphosphonates in the treatment of osteoporosis. BMJ.

[10] Russell RG, Watts NB, Ebetino FH, Rogers MJ (2008). Mechanisms of action of bisphosphonates: similarities and differences and their potential influence on clinical efficacy. Osteoporos. Int..

[11] Wells, G. A. *et al*. Etidronate for the primary and secondary prevention of osteoporotic fractures in postmenopausal women. *Cochrane Database of Systematic Reviews* Issue 1. Art. No.: CD003376, doi:10.1002/14651858.CD003376.pub3 (2008). 10.1002/14651858.CD003376.pub3 PMC6999803 18254018

[12] Lambrinoudaki I, Vlachou S, Galapi F, Papadimitriou D, Papadias K (2008). Once-yearly zoledronic acid in the prevention of osteoporotic bone fractures in postmenopausal women. Clin. Interv. Aging.

[13] Gong L, Altman RB, Klein TE (2011). Bisphosphonates pathway. Pharmacogenet. Genomics.

[14] Sparidans RW, Twiss IM, Talbot S (1998). Bisphosphonates in bone diseases. Pharm. World Sci..

[15] Kennel KA, Drake MT (2009). Adverse effects of bisphosphonates: Implications for osteoporosis management. Mayo Clin. Proc..

[16] Wang, L., Zhang, M., Yang, Z. & Xu, B. The first pamidronate containing polymer and copolymer. *Chem. Commun*. 2795–2797 (2006). 10.1039/b605365c 17009466

[17] Huang W, Wang Y, Ren L, Du C, Shi X (2009). A novel PHBV/HA microsphere releasing system loaded with alendronate. Mater. Sci. Engin..

[18] Chaudhari KR (2012). Bone metastasis targeting: A novel approach to reach bone using Zoledronate anchored PLGA nanoparticle as carrier system loaded with Docetaxel. J. Controlled Release.

[19] Sundell MJ, Ekman KB, Svarfvar BL, Näsman JH (1995). Preparation of poly[ethylene-g(vinylbenzyl chloride)] and functionalization with bis(phosphonic acid) derivatives. React. Polym..

[20] Johnston TP, Webb CL, Schoen F-J, Levy RJ (1993). Site-specific delivery of ethanehydroxy diphosphonate from refillable polyurethane reservoirs to inhibit bioprosthetic tissue calcification. J. Controlled Release.

[21] Kim CW (2010). *In situ* fabrication of alendronate-loaded calcium phosphate microspheres: controlled release for inhibition of osteoclastogenesis. J. Control Release.

[22] Tran, R. T., Gyawali, D., Nair, P., Yang, J. Biodegradable Injectable Systems for Bone Tissue Engineering, RSC Green Chemistry No. 12, A Handbook of Applied Biopolymer Technology: Synthesis, Degradation and Applications, Edited by Sharma, S. K. and Mudhoo, A. Royal Society of Chemistry, Chapter 14, pp. 419–451 (2011).

[23] Cha C (2014). Microfluidics-Assisted Fabrication of Gelatin-Silica Core−Shell Microgels for Injectable Tissue Constructs. Biomacromolecules.

[24] He Q, Zhang Z, Gao Y, Shi J, Li Y (2009). Intracellular Localization and Cytotoxicity of Spherical Mesoporous Silica Nano- and Microparticles. Small.

[25] Croissant JG, Fatieiev Y, Khashab NM (2017). Degradability and Clearance of Silicon, Organosilica, Silsesquioxane, Silica Mixed Oxide, and Mesoporous Silica Nanoparticles. Adv. Mater..

[26] He QJ, Shi JL, Zhu M, Chen Y, Chen F (2010). The three-stage *in vitro* degradation behavior of mesoporous silica in simulated body fluid. Microporous Mesoporous Mater..

[27] Carlisle EM (1970). Silicon: a possible factor in bone calcification. Science.

[28] Sharif F, Porta F, Meijer AH, Kros A, Richardson MK (2012). Mesoporous silica nanoparticles as a compound delivery system in zebrafish embryos. Int. J. Nanomed..

[29] Whang S (2009). Ordered mesoporous materials for drug delivery. Microporous Mesoporous Mater..

[30] Balas F, Manzano M, Horcajada P, Vallet-Regí M (2006). Confinement and controlled release of bisphosphonates on ordered mesoporous silica-based materials. J. Am. Chem. Soc..

[31] Nancollas GH (2006). Novel insights into actions of bisphosphonates on bone: differences in interactions with hydroxyapatite. Bone.

[32] Silvestre J-P, Dao NQ, Leroux Y (2001). A survey of the behavior of the hydroxybisphosphonic function in crystallized acids, metallic salts, and some related compounds. Heteroatom Chem..

[33] Alanne A-L (2012). Systematic study of the physicochemical properties of a homologous series of aminobisphosphonates. Molecules.

[34] Alanne A-L (2013). A novel bisphosphonate-based solid phase method for effective removal of chromium(III) from aqueous solutions and tannery effluents. RSC-Adv..

[35] Bibent, N. *et al*. Solid-State NMR Spectroscopic Studies of Propylphosphonic Acid Functionalized SBA-15 Mesoporous Silica: Characterization of Hydrogen-Bonding Interactions. *Eur. J. Inorg. Chem*. 2350–2361 (2013).

[36] Ironside MS, Duer MJ, Reid DG, Byard S (2010). Bisphosphonate protonation states, conformations, and dynamics on bone mineral probed by solid-state NMR without isotope enrichment. Eur. J. Pharm. Biopharm..

[37] Meloun M, Ferenčíková Z, Netolická L, Pekárek T (2011). Thermodynamic Dissociation Constants of Alendronate and Ibandronate by Regression Analysis of Potentiometric Data. J. Chem. Eng. Data.

[38] Boichenko AP, Markov VV, Kong HL, Matveeva AG, Loginova LP (2009). Re-evaluated data of dissociation constants of alendronic, pamidronic and olpadronic acids. Cent. Eur. J. Chem..

[39] Bogomilova A, Hägele G, Troev K, Wagner E, Günther M (2012). Hydrogen bonding in α-aminophosphonic acids. Phosphorus Sulfur Silicon.

[40] Egorov, M. *et al*. A one-pot synthesis of 1-hydroxy-1,1-bis(phosphonic acid)s starting from the corresponding carboxylic acids. *Eur. J. Org. Chem*. 7148–7154 (2011).

[41] Massiot D (2002). Modelling one- and two-dimensional solid-state NMR spectra. Magn. Reson. Chem..

