# Supplementary material for: Smart, programmable and responsive injectable hydrogels for controlled release of cargo osteoporosis drugs
Source: Sci Rep. 2017 Jul 6;7:4743. doi: 10.1038/s41598-017-04956-3 (PMC5500573; doi:10.1038/s41598-017-04956-3)
Supplement: Supplementary file 2 — Supplementary Information [file 41598_2017_4956_MOESM2_ESM.pdf]

Supplementary Information

for

Smart, programmable and responsive injectable hydrogels for controlled release of cargo osteoporosis drugs

by

K.E. Papathanasiou, P. Turhanen, S.I. Brückner,

E. Brunner, and K.D. Demadis

Table of Contents

(1) Bisphosphonate description (BP notation and structures)
(2) Experimental Section (experimental details, instruments, synthesis, spectra)
(3) NMR spectra of selected bisphosphonates
(4) Gel fabrication and characterization
(5) Miscellaneous controlled release comparative data
(6) Mathematical data treatment
(7) Gel NMR (^{31}P MAS and ^{1}H-^{31}P CP MAS) spectra

1. **Bisphosphonate description**

Table S-1. Bisphosphonate notation and structures

**Bisphosphonate common name**	**Bisphosphonate Chemical name**	**Bisphosphonate abbreviation**	**Bisphosphonate structure**	**Available as "acid"**	**Available as "salt"**
**Etidronic acid**	1-hydroxyethane-1,1-bisphosphonic acid	***ETID***	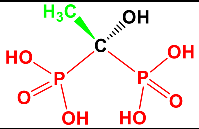	YES	Tetrasodium salt
**Not available**	1-hydroxybutane-1,1-bisphosphonic acid disodium salt	***C3BP***	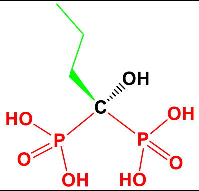	NO	Disodium salt
**Not available**	1-hydroxyhexane-1,1-bisphosphonic acid disodium salt	***C5BP***	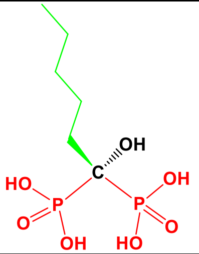	NO	Disodium salt
**Pamidronic acid**	3-Amino-1-hydroxypropane-1,1-diphosphonic acid	***PAM***	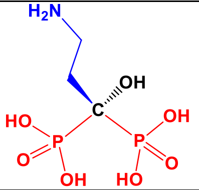	YES	NO
**Alendronic acid**	3-Amino-1-hydroxybutane-1,1-diphosphonic acid	***ALE***	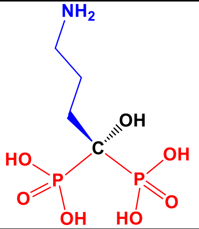	YES	Disodium salt
**Not available**	4-Amino-1-hydroxypentane-1,1-diphosphonic acid	***C4NBP***	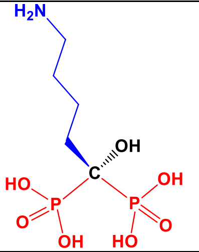	YES	NO
**Neridronic acid**	6-Amino-1-hydroxyhexane-1,1-bisphosphonic acid	***NER***	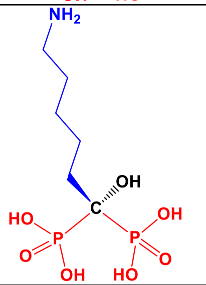	YES	NO

2. **Experimental Section**

Materials. Sodium silicate pentahydrate, $Na_2SiO_3{\cdot}5H_2O$, silicic acid (< 20 micron, refined, 99.9%) and potassium hydroxide was purchased from Sigma Aldrich. Rubidium hydroxide hydrate was purchased from Alfa-Aesar. ETID (either as solid tetrasodium salt or as acid in aqueous solution) was used as received from Solutia Inc. Deuterium oxide (99.9 atom % D) that contained 0.05 wt. % 3-(trimethylsilyl)propionic-2,2,3,3-d4 acid, sodium salt purchased also from Sigma Aldrich. Deionized water from an ion-exchange resin was used for all experiments and stock solution preparations.

Instrumentation. Solid state NMR experiments were performed on a Bruker Avance 300 NMR spectrometer with a 7 mm MAS wide bore probe. The operating resonance frequency (rf) of 300.1 MHz for ^{1}H, 59.6 MHz for ^{29}Si and of 121.5 MHz for ^{31}P measurements. 7 mm Zirconia rotors with KEL-F inserts were used. For direct excitation measurements π/2 pulses at rf fields of 34.7 kHz on ^{29}Si and 65.8 kHz on 31P were applied. The interscan delay was set to 180 s for ^{31}P and 120 s for ^{29}Si. During acquisition TPPM decoupling with rf field at 50.0 kHz was used. Measurements were performed at a sample spinning speed of 7 kHz. For the ^{1}H-^{31}P CP MAS experiments a ramped CP by using an rf field of 53.2 kHz for the spinlock on ^{31}P, an 80%-100% ramp on the proton channel, and a contact time of 1.5 ms were applied. The corresponding decoupling was performed using the TPPM decoupling scheme at an rf field of 50.0 kHz during acquisition. The interscan delay was set to 5 s. An AVANCE 300 (Bruker, Karlsruhe, Germany) spectrometer was used for the BP release experiments. SEM data and images collected with a JOEL JSM-6390LV electron microscope.

General comments on the synthesis of bisphosphonates. Alendronate and neridronate were synthesized and characterized as reported elsewhere [A.-L. Alanne, H. Hyvönen, M. Lahtinen, M. Ylisirniö, P. Turhanen, E. Kolehmainen, S. Peräniemi, J. Vepsäläinen, Molecules, 2012, 17, 10928-10945]. C3BP (disodium salt) and C5BP (disodium salt) were synthesized according to the procedure described Egorov *et al.* [M.

Egorov, S. Aoun, M. Padrines, F. Redini, D. Heymann, J. Lebreton, M. Mathé-Allainmat, Eur. J. Org. Chem. 2011, 7148–7154] and can be found in detail with NMR and HRMS characterization data in supporting information.

Preparation of gels. Herein, the synthesis of an ETID-loaded gel is described, as an example. All other BP-loaded gels were prepared in the same manner. The synthesis of each BP-loaded gel was repeated four (4) times using identical shape and diameter borosilicate glass beakers. In a beaker 10 mL of DI water was added. In this a quantity (0.66 g, 3.14 mmol) of sodium metasilicate pentathydrate was dissolved, together with 0.50 g, 1.70 mmol) of tetrasodium ETID, while keeping the solution under stirring. The pH value of this solution was ~ 12.5. The pH was adjusted to 7.00 with the use 0.75 mL of concentrated HCl (37 %). This particular pH value was selected because the polymerization of silicic acid has the highest rate there. Gel formation commences within 10 minutes, however the freshly formed and "loose" gel was allowed to mature for 12 hours, after which a shapely and translucent gel formed. Gel preparation can be reproducibly repeated and can be modified by altering the amount of Na^+ ions, replacing the alkali ion, or changing the entrapped BP.

BP-containing gels for all remaining BPs were prepared in the same manner, using quantities shown in the Table below.

BP	Mass (g)	mmoles
**ETID**	0.50 g	1.70 mmol
**C3BP**	0.38 g	1.36 mmol
**C5BP**	0.48 g	1.56 mmol
**PAM**	0.29 g	1.22 mmol
**ALE**	0.37 g	1.25 mmol
**C4NBP**	0.35 g	1.31 mmol
**NER**	0.35 g	1.25 mmol

Controlled release of BPs from gels. On top of the solidified gel (see above), a volume of deionized (DI) water (50 mL), pre-acidified to pH ~ 3 was carefully poured. This marked the initiation of the controlled release process (t = 0), which continued for

48 hours. For the initial 6-hour period an aliquot of 0.350 mL was withdrawn from the supernatant every hour. After the 6th hour and for the next 12 hours, sampling was performed every 3 hours. Finally, after the 18th hour and until the end of the release experiment (at the 48th hour) sampling was performed every 8 hours. The withdrawn samples were mixed with 0.150 mL of deuterium oxide (99.9 atom % D) that contained 0.05 wt. % (4.3375 μmol) 3-(trimethylsilyl)propionic-2,2,3,3-d_4 acid, sodium salt, TSP) as standard. ^{1}H NMR spectra were recorded on a Bruker AVANCE 300 MHz NMR (Bruker, Karlsruhe, Germany) spectrometer at 293.2 K operating at a proton NMR frequency of 300.13 MHz. Standard solvent (D_2O) was used as internal lock. Each ^{1}H spectrum consisted of 32 scans requiring 3 min. and 39 min. acquisition time with the following parameters: Spectral width = 20.5671 μs, pulse width (P1) = 15.000 μs, , and relaxation delay (D1) = 4.000 seconds. Polynomial 4th-order baseline correction was performed before manual integration of all NMR spectra. Proton and carbon chemical shifts in D_2O are reported relative to TSP. The characteristic peaks for each compound were integrated using the integration tool available from the Bruker software (TopSpin 3.2). For each compound we selected the integration value of the sharpest peak. All the integration values were cross-checked in order to ensure the best result for each compound (see spectra in NMR spectra, below).

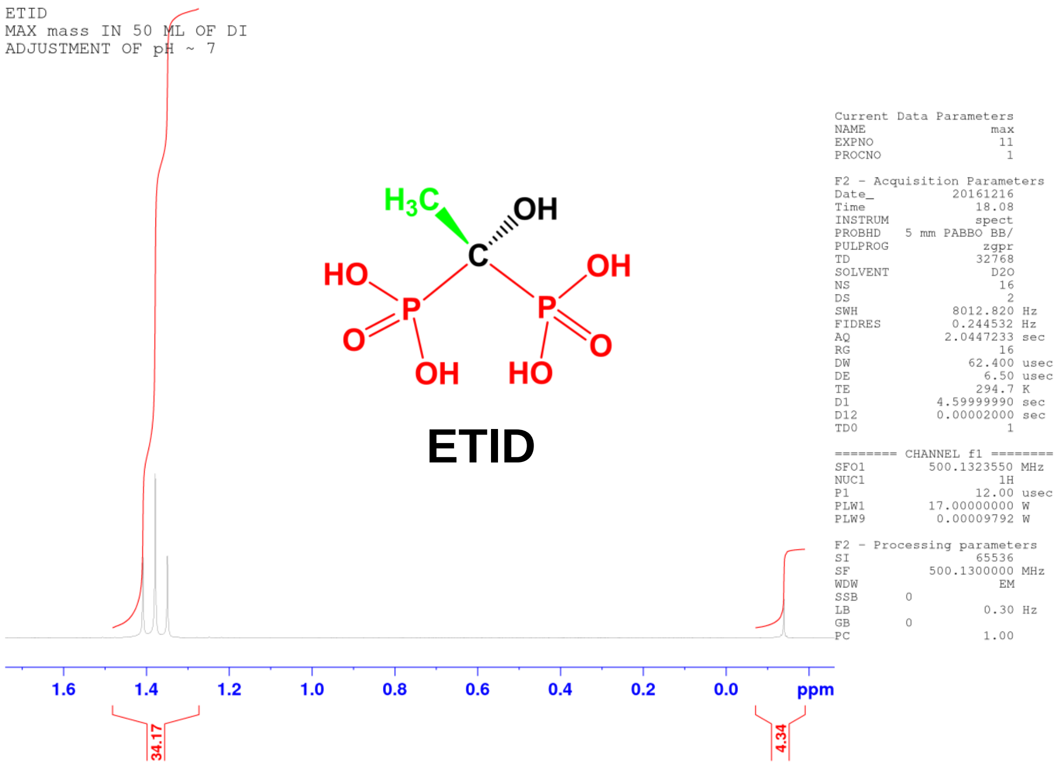
ETID
MAX mass IN 50 ML OF DI
ADJUSTMENT OF pH ~ 7
H3C
OH
C
HO
P
O
OH
P
OH
O
OH
HO
ETID
Current Data Parameters
NAME max
EXPNO 11
PROCNO 1
F2 - Acquisition Parameters
Date_ 20161216
Time 18.08
INSTRUM spect
PROBHD 5 mm PABBO BB/
PULPROG zgpr
TD 32768
SOLVENT D2O
NS 16
DS 2
SWH 8012.820 Hz
FIDRES 0.244532 Hz
AQ 2.0447233 sec
RG 16
DW 62.400 usec
DE 6.50 usec
TE 294.7 K
D1 4.59999990 sec
D12 0.00002000 sec
TD0 1
======== CHANNEL f1 ========
SFO1 500.1323550 MHz
NUC1 1H
P1 12.00 usec
PLW1 17.00000000 W
PLW9 0.00009792 W
F2 - Processing parameters
SI 65536
SF 500.1300000 MHz
WDW EM
SSB 0
LB 0.30 Hz
GB 0
PC 1.00
1.6
1.4
1.2
1.0
0.8
0.6
0.4
0.2
0.0
ppm
34.17
4.34

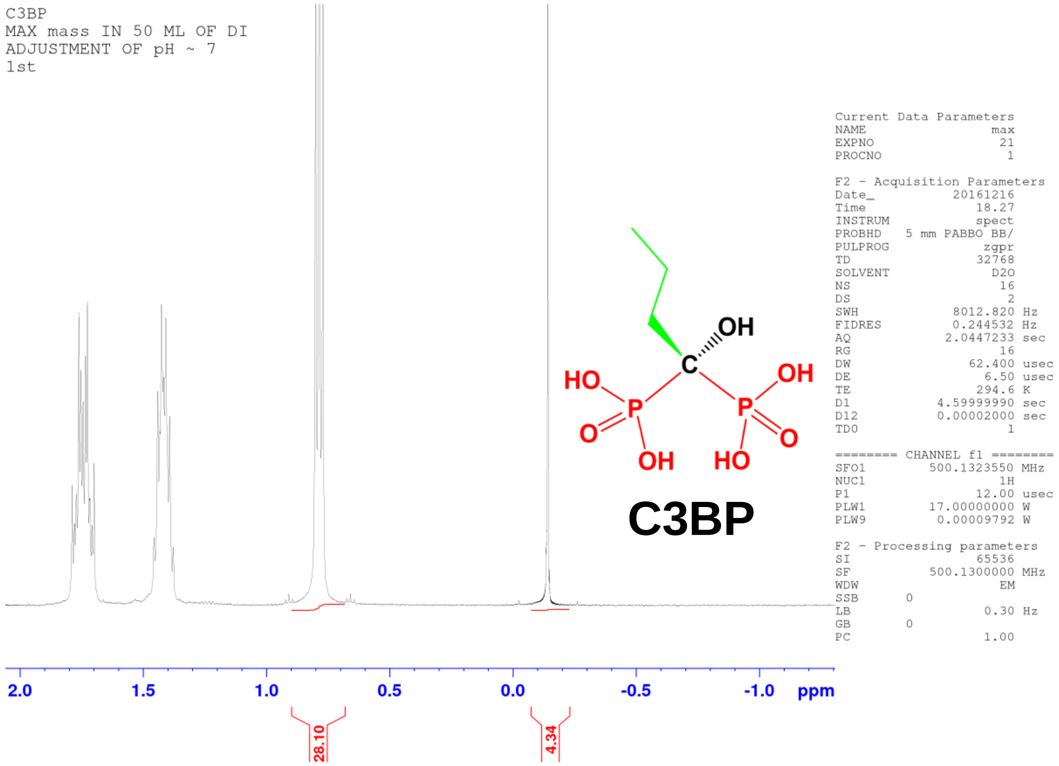
C3BP
MAX mass IN 50 ML OF DI
ADJUSTMENT OF pH ~ 7
1st
OH
C
HO
P
O
OH
P
OH
O
HO
C3BP
Current Data Parameters
NAME max
EXPNO 21
PROCNO 1
F2 - Acquisition Parameters
Date_ 20161216
Time 18.27
INSTRUM spect
PROBHD 5 mm PABBO BB/
PULPROG zgpr
TD 32768
SOLVENT D2O
NS 16
DS 2
SWH 8012.820 Hz
FIDRES 0.244532 Hz
AQ 2.0447233 sec
RG 16
DW 62.400 usec
DE 6.50 usec
TE 294.6 K
D1 4.59999990 sec
D12 0.00002000 sec
TD0 1
======== CHANNEL f1 ========
SFO1 500.1323550 MHz
NUC1 1H
P1 12.00 usec
PLW1 17.00000000 W
PLW9 0.00009792 W
F2 - Processing parameters
SI 65536
SF 500.1300000 MHz
WDW EM
SSB 0
LB 0.30 Hz
GB 0
PC 1.00
2.0
1.5
1.0
0.5
0.0
-0.5
-1.0
ppm
28.10
4.34

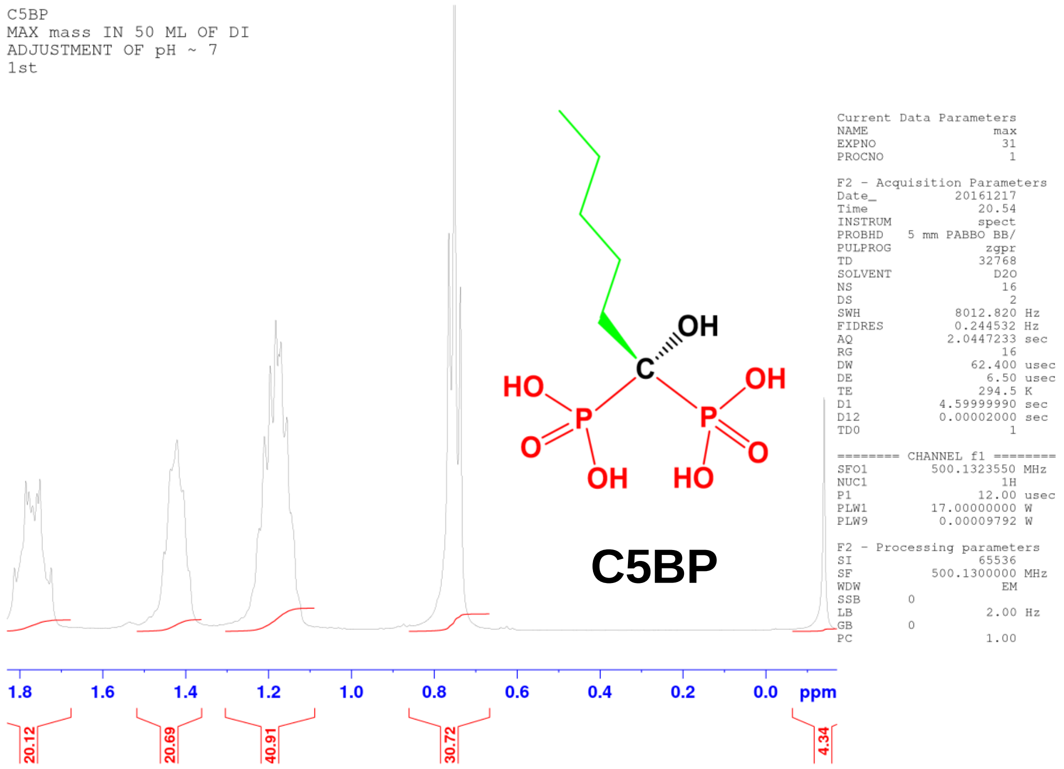
C5BP
MAX mass IN 50 ML OF DI
ADJUSTMENT OF pH ~ 7
1st
Current Data Parameters
NAME max
EXPNO 31
PROCNO 1
F2 - Acquisition Parameters
Date_ 20161217
Time 20.54
INSTRUM spect
PROBHD 5 mm PABBO BB/
PULPROG zgpr
TD 32768
SOLVENT D2O
NS 16
DS 2
SWH 8012.820 Hz
FIDRES 0.244532 Hz
AQ 2.0447233 sec
RG 16
DW 62.400 usec
DE 6.50 usec
TE 294.5 K
D1 4.59999990 sec
D12 0.00002000 sec
TD0 1
======== CHANNEL f1 ========
SFO1 500.1323550 MHz
NUC1 1H
P1 12.00 usec
PLW1 17.00000000 W
PLW9 0.00009792 W
F2 - Processing parameters
SI 65536
SF 500.1300000 MHz
WDW EM
SSB 0
LB 2.00 Hz
GB 0
PC 1.00
OH
HO
OH
C
P
P
O
O
OH
HO
C5BP
1.8
1.6
1.4
1.2
1.0
0.8
0.6
0.4
0.2
0.0
ppm
20.12
20.69
40.91
30.72
4.34

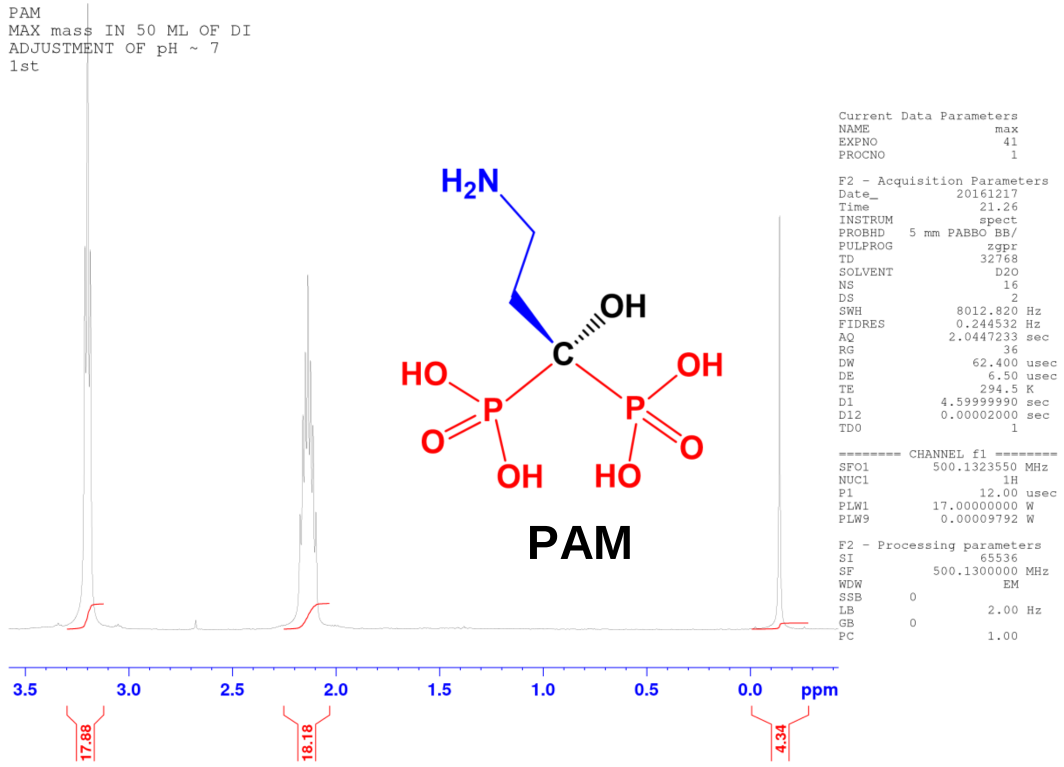
PAM
MAX mass IN 50 ML OF DI
ADJUSTMENT OF pH ~ 7
1st
Current Data Parameters
NAME max
EXPNO 41
PROCNO 1
F2 - Acquisition Parameters
Date_ 20161227
Time 21.26
INSTRUM spect
PROBHD 5 mm PABBO BB/
PULPROG zgpr
TD 32768
SOLVENT D2O
NS 16
DS 2
SWH 8012.820 Hz
FIDRES 0.244532 Hz
AQ 2.0447233 sec
RG 36
DW 62.400 usec
DE 6.50 usec
TE 294.5 K
D1 4.59999990 sec
D12 0.00002000 sec
TD0 1
======== CHANNEL f1 ========
SFO1 500.1323550 MHz
NUC1 1H
P1 12.00 usec
PLW1 17.00000000 W
PLW9 0.00009792 W
F2 - Processing parameters
SI 65536
SF 500.1300000 MHz
WDW EM
SSB 0
LB 2.00 Hz
GB 0
PC 1.00
H2N
OH
HO
OH
C
P
P
O
O
OH
HO
PAM
3.5
3.0
2.5
2.0
1.5
1.0
0.5
0.0
ppm
17.88
18.18
4.34

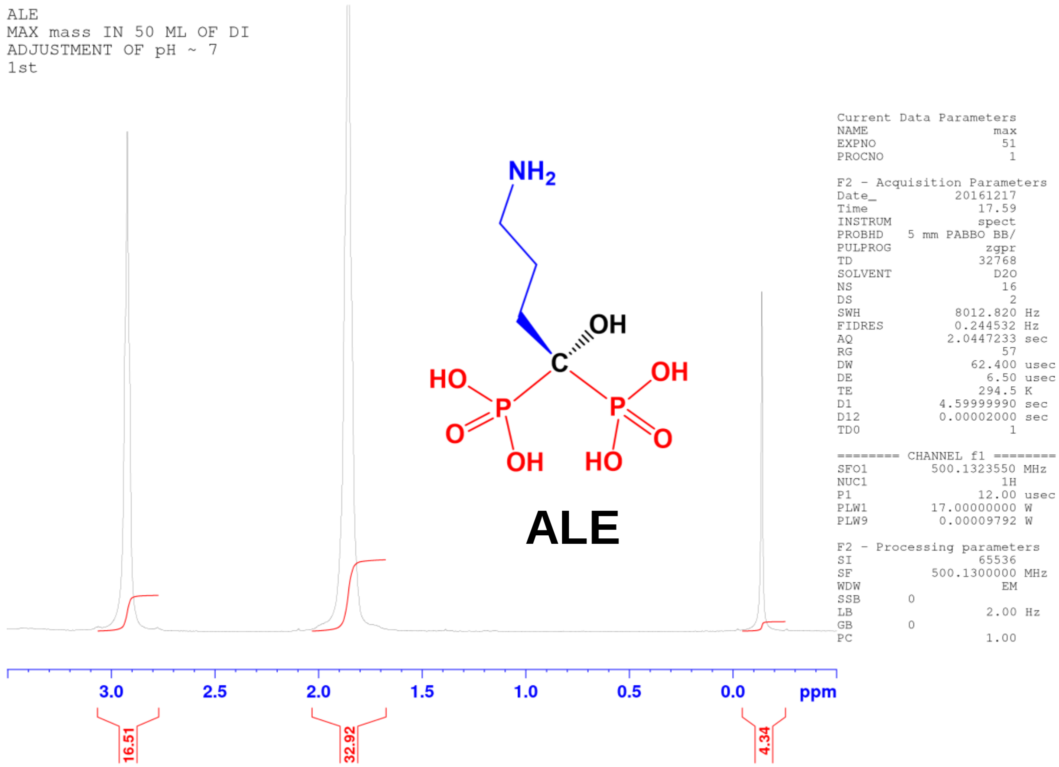
ALE
MAX mass IN 50 ML OF DI
ADJUSTMENT OF pH ~ 7
1st
NH2
OH
C
HO
OH
P
P
O
O
OH
HO
ALE
Current Data Parameters
NAME max
EXPNO 51
PROCNO 1
F2 - Acquisition Parameters
Date_ 20161217
Time 17.59
INSTRUM spect
PROBHD 5 mm PABBO BB/
PULPROG zgpr
TD 32768
SOLVENT D2O
NS 16
DS 2
SWH 8012.820 Hz
FIDRES 0.244532 Hz
AQ 2.0447233 sec
RG 57
DW 62.400 usec
DE 6.50 usec
TE 294.5 K
D1 4.59999990 sec
D12 0.00002000 sec
TD0 1
======== CHANNEL f1 ========
SFO1 500.1323550 MHz
NUC1 1H
P1 12.00 usec
PLW1 17.00000000 W
PLW9 0.00009792 W
F2 - Processing parameters
SI 65536
SF 500.1300000 MHz
WDW EM
SSB 0
LB 2.00 Hz
GB 0
PC 1.00
3.0
2.5
2.0
1.5
1.0
0.5
0.0
ppm
16.51
32.92
4.34

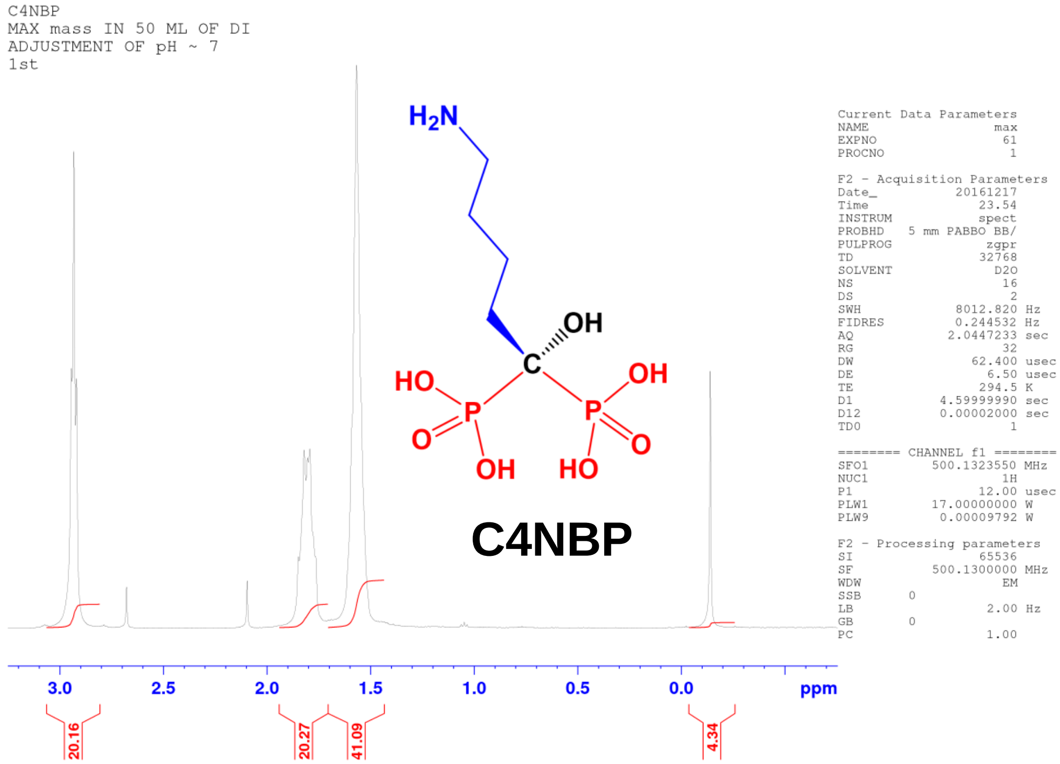
C4NBP
MAX mass IN 50 ML OF DI
ADJUSTMENT OF pH ~ 7
1st
H2N
OH
C
HO
OH
P
P
O
O
OH
HO
C4NBP
Current Data Parameters
NAME max
EXPNO 61
PROCNO 1
F2 - Acquisition Parameters
Date_ 20161217
Time 23.54
INSTRUM spect
PROBHD 5 mm PABBO BB/
PULPROG zgpr
TD 32768
SOLVENT D2O
NS 16
DS 2
SWH 8012.820 Hz
FIDRES 0.244532 Hz
AQ 2.0447233 sec
RG 32
DW 62.400 usec
DE 6.50 usec
TE 294.5 K
D1 4.59999990 sec
D12 0.00002000 sec
TD0 1
======== CHANNEL f1 ========
SFO1 500.1323550 MHz
NUC1 1H
P1 12.00 usec
PLW1 17.00000000 W
PLW9 0.00009792 W
F2 - Processing parameters
SI 65536
SF 500.1300000 MHz
WDW EM
SSB 0
LB 2.00 Hz
GB 0
PC 1.00
3.0
2.5
2.0
1.5
1.0
0.5
0.0
ppm
20.16
20.27
41.09
4.34

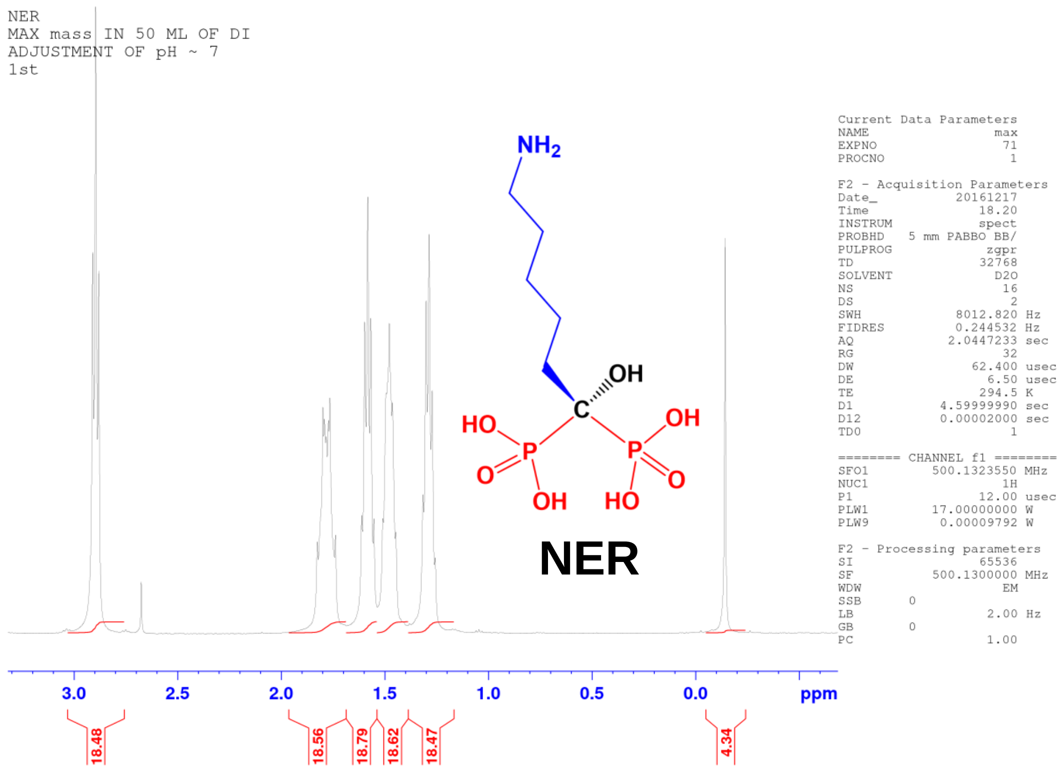

Syntheses of selected bisphosphonates

General

^{1}H and ^{31}P NMR spectra were recorded on a 600 MHz spectrometer operating at 600.2 and 243.0 MHz, respectively; ^{13}C NMR spectra were recorded on a 500 MHz spectrometer operating at 125.8 MHz. The solvent residual peak was used as a standard for ^{1}H measurements in D_2O (4.79 ppm) and in ^{13}C measurements CD_3OD were added to be as a reference (49.00 ppm) [Gottlieb, H.E.; Kotlyar, V.; Nudelman, A. NMR Chemical Shifts of Common Laboratory Solvents as Trace Impurities. *J. Org. Chem.* **1997**, *62*, 7512-7515]. 85% H_3PO_4 was used as an external standard in the ^{31}P measurements. The $^{n}J_{HH}$ couplings were calculated from proton spectra and all J values are given in Hz. The $^{n}J_{CP}$ couplings were calculated from carbon spectra with the coupling constants given in parenthesis as Hz. Mass spectra were recorded with a Finnigan LCQ quadrupole ion trap mass spectrometer (Finnigan MAT, San Jose, CA, USA) equipped with an electrospray ionization source. The purity of the products was determined from ^{1}H and ^{31}P NMR spectra and was ≥ 95 % unless stated otherwise.

Synthesis of 1-hydroxybutane-1,1-bisphosphonic acid disodium salt (C3BP) 1 M catecholborane solution in THF (36.5 mL, 36.5 mmol) was added to a flask containing butyric acid (3.2 g, 36.3 mmol) under an nitrogen atmosphere at room temperature. The mixture was stirred for about 1 hour until no more gas evolution was observed. Tris(trimethylsilyl) phosphite (22.3 g, 25.0 mL, 74.8 mmol, 2.05 equiv.) was added and stirring was continued for 20 h. Methanol (120 mL) was added and, after stirring for 4-5 h., the solvents were evaporated *in vacuo*. The residue was dissolved in water (15 mL) and about 75 mL of MeOH was added. pH was adjusted to 8-9 by adding 40% NaOH with stirring. White precipitate formed and after 0.5 h it was filtered, washed with H_2O/MeOH (1:5) and dried *in vacuo* for several days with warming (MeOH was extremely “tightly” in crystal product). 1-hydroxybutane-1,1-bisphosphonic acid disodium salt (6.01 g, 60%) was obtained as a white powder. ^{1}H NMR (D_2O): δ 1.88-1.79 (m, 2H, CH_2), 1.56-1.47 (m, 2H, CH_2), 0.89 (t, 3H, ^3J = 7.4). ^{13}C NMR (D_2O, CD_3OD as ref.) δ 75.4 (t, $^1J_{CP}$ = 130.8, P-*C*-P), 37.2, 18.2 (t, $^2J_{CP}$ = 6.3), 15.2. ^{31}P NMR (D_2O) δ 18.79. MS (ESI$^-$) calcd. for $C_4H_{11}O_7P_2^-$ [M-H]$^-$ 232.9986, found: 232.9985.

Synthesis of 1-hydroxyhexane-1,1-bisphosphonic acid disodium salt (C5BP). Prepared similarly to compound **C3BP** from hexanoic acid (4.23 g, 36.4 mmol), 1 M cathecolborane solution in THF (36.4 mL, 36.4 mmol) and tris(trimethylsilyl) phosphite (22.3 g, 25.0 mL, 74.8 mmol, 2.05 equiv.). 1-hydroxyhexane-1,1-bisphosphonic acid disodium salt (7.34 g, 66%) was obtained as a white powder. ^{1}H NMR (D_2O): δ 1.92-1.83 (m, 2H, CH_2), 1.57-1.50 (m, 2H, CH_2), 1.36-1.24 (m, 4H, 2 x CH_2), 0.87 (t, 3H, ^3J = 7.2). ^{13}C NMR (D_2O, CD_3OD as ref.) δ 75.3 (t, $^1J_{CP}$ = 132.0, P-*C*-P), 34.9, 33.1, 24.4 (t, $^2J_{CP}$ = 5.7), 22.9,14.4. ^{31}P NMR (D_2O) δ 18.72. MS (ESI$^-$) calcd. for $C_6H_{15}O_7P_2^-$ [M-H]$^-$ 261.0299, found: 261.0297.

3. NMR spectra of selected bisphosphonates

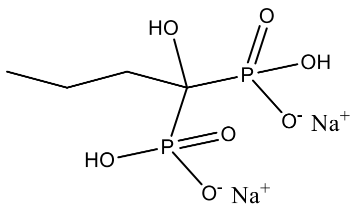

C3BP

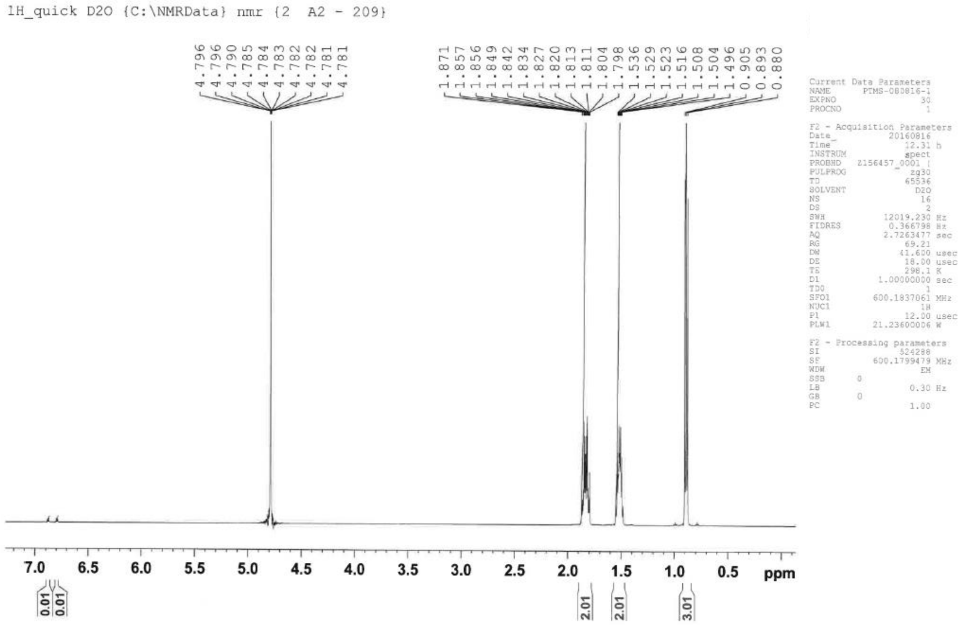

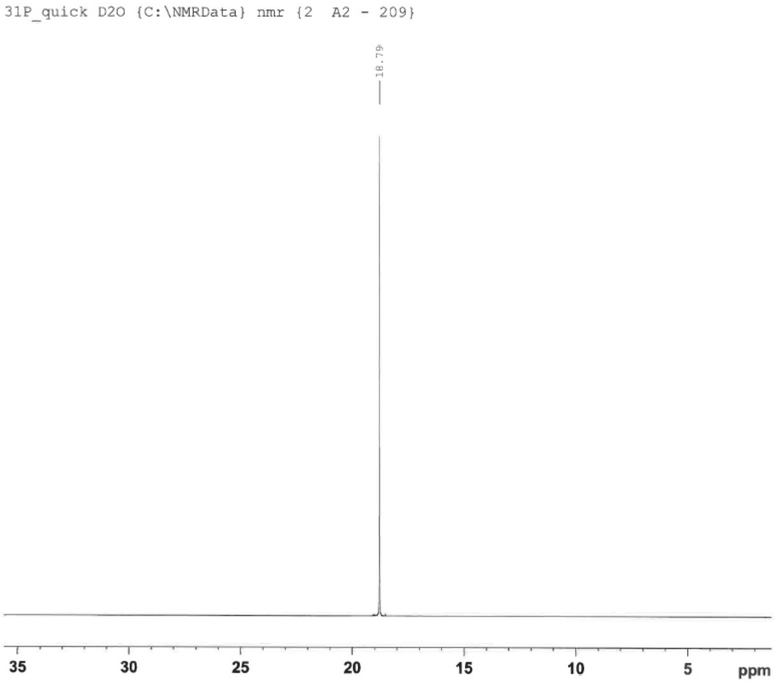

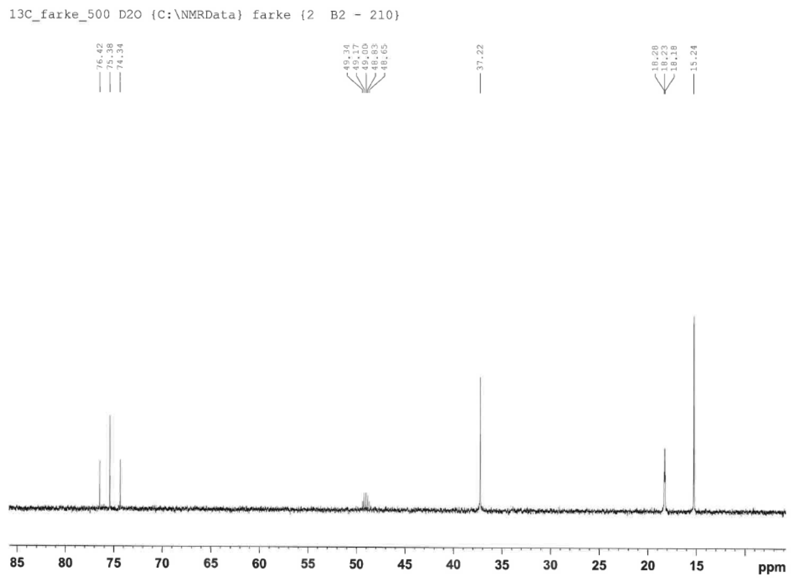
13C_farke_500 D2O {C:\NMRData} farke {2 B2 - 210}
ppm

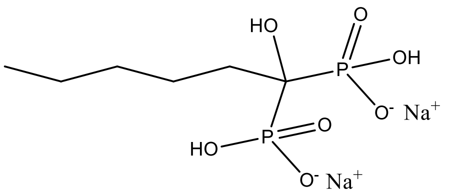
HO
O
OH
O- Na+
O
HO
O- Na+

C5BP

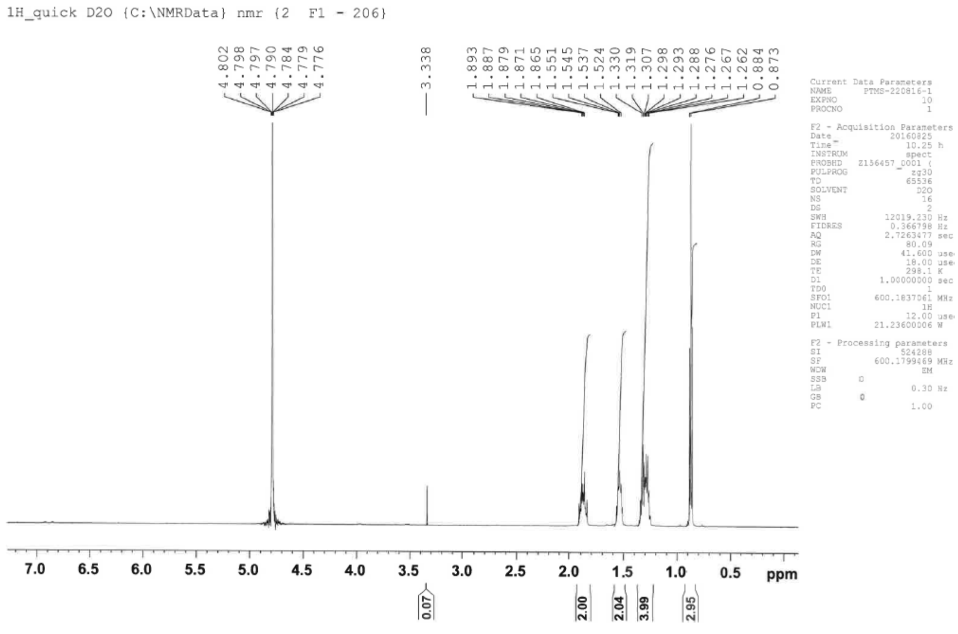
1H_quick D2O {C:\NMRData} nmr {2 F1 - 206}
ppm

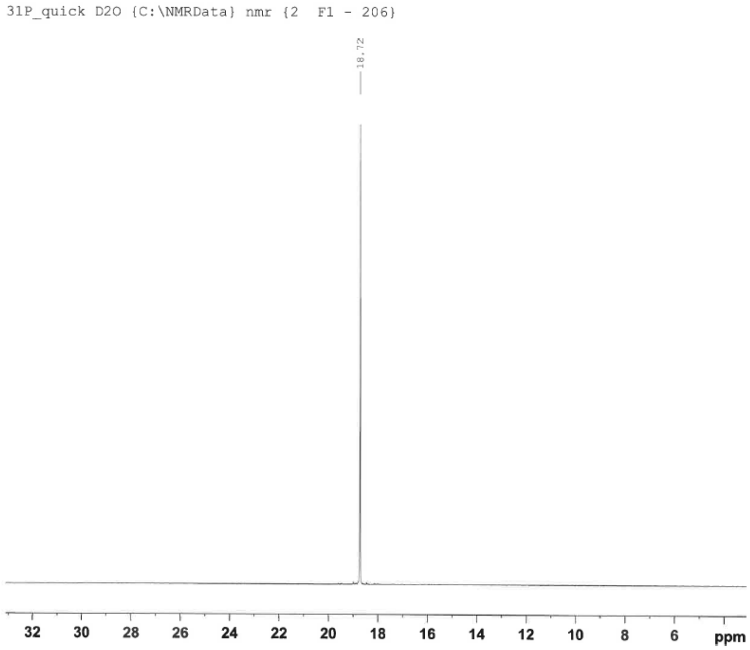
31P_quick D2O {C:\NMRData} nmr {2 F1 - 206}
18.72
32 30 28 26 24 22 20 18 16 14 12 10 8 6 ppm

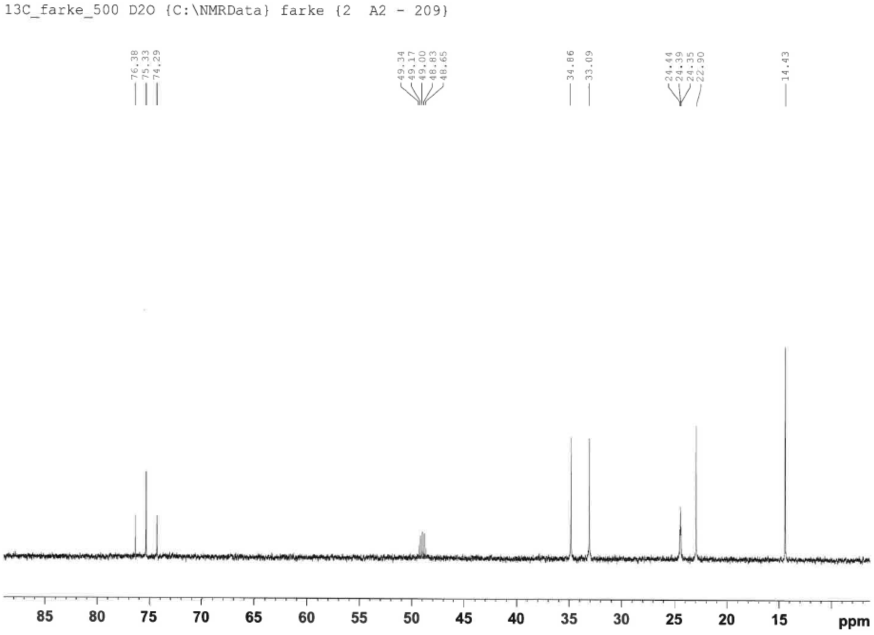
13C_farke_500 D2O {C:\NMRData} farke {2 A2 - 209}
76.38
75.33
74.29
49.34
49.17
49.00
48.83
48.65
34.86
33.09
24.44
24.39
24.35
22.90
14.43
85 80 75 70 65 60 55 50 45 40 35 30 25 20 15 ppm

4. **Gel fabrication and characterization**

Images of injectable gels

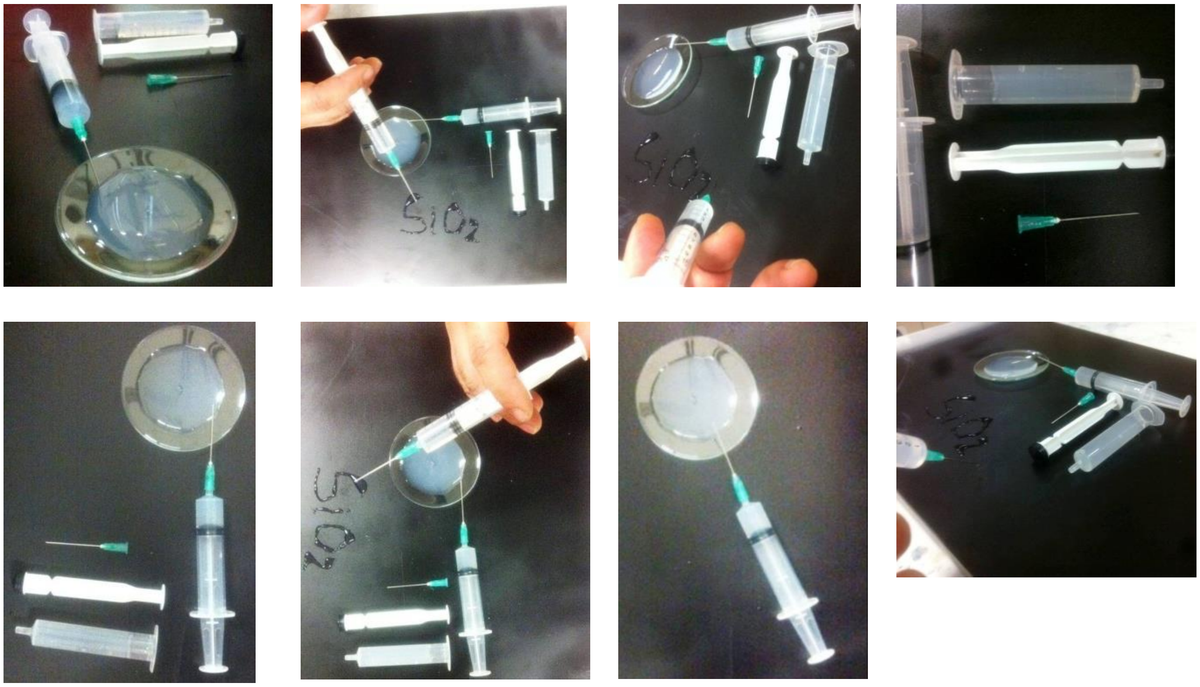

Figure S-1. The prepared gels are injectable.

SEM characterization and imaging of “empty” and “drug-loaded” gels

Samples of all studied hydrogels (“empty”, ETID-loaded, and “emptied gels) were treated several times and in series with Ethanol/H_2O solutions, 30/70, 50/50, 70/30, 90/10, and finally 100/0, in order to achieve complete dehydration. Then a typical protocol of CPD was followed before the SEM studies.

“Empty” gels (no bisphosphonate present)

Gel surface (1st sample)

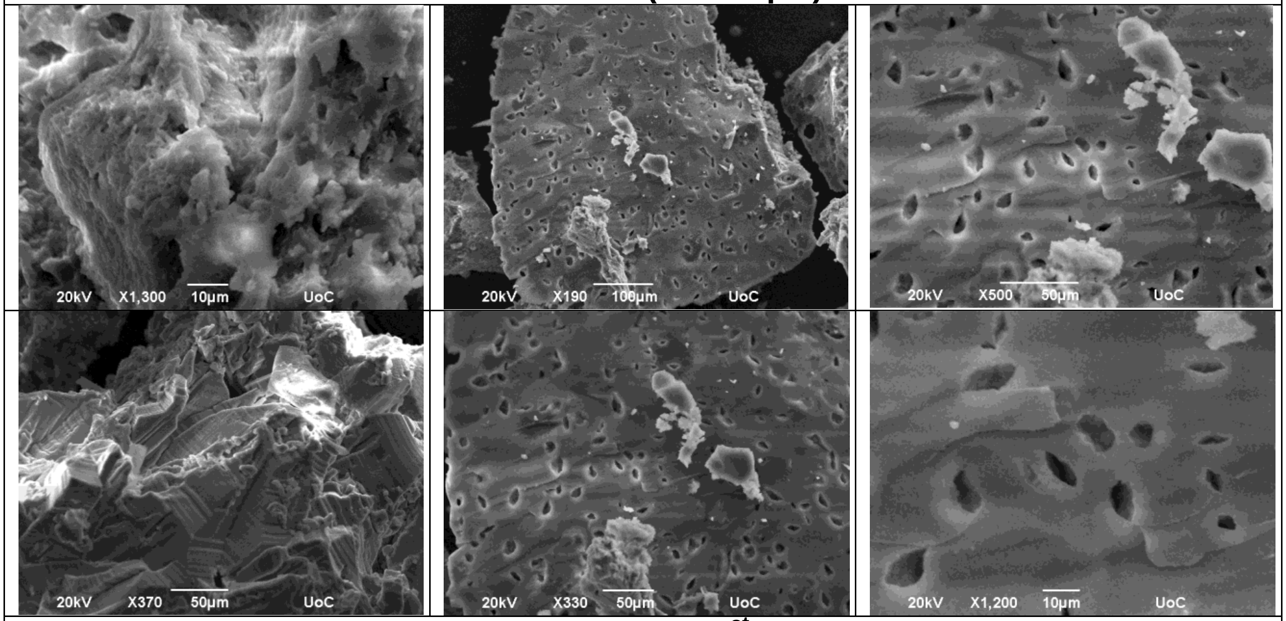

Gel cross-sections (1st sample)

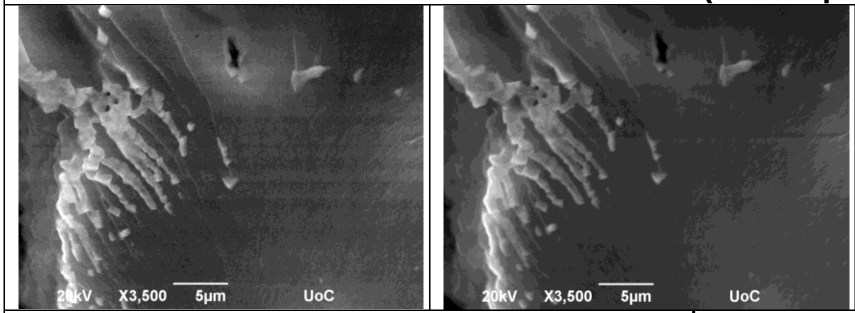

Gel surface (2nd sample)

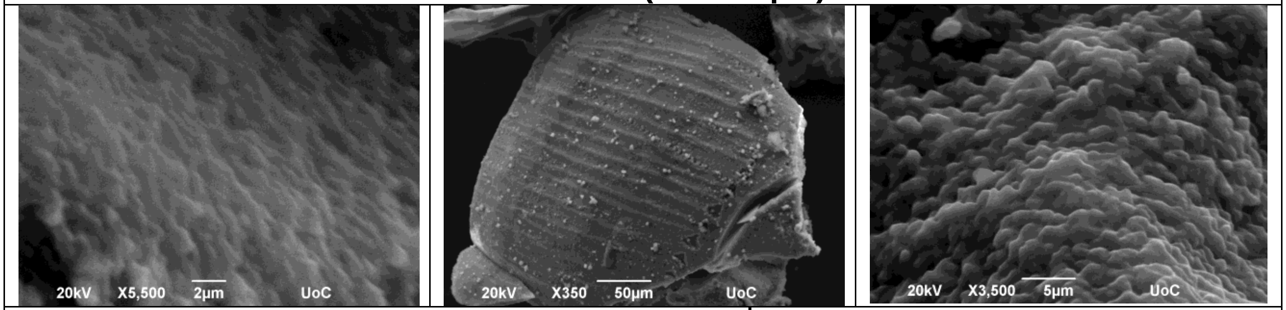

Gel cross-sections (2nd sample)

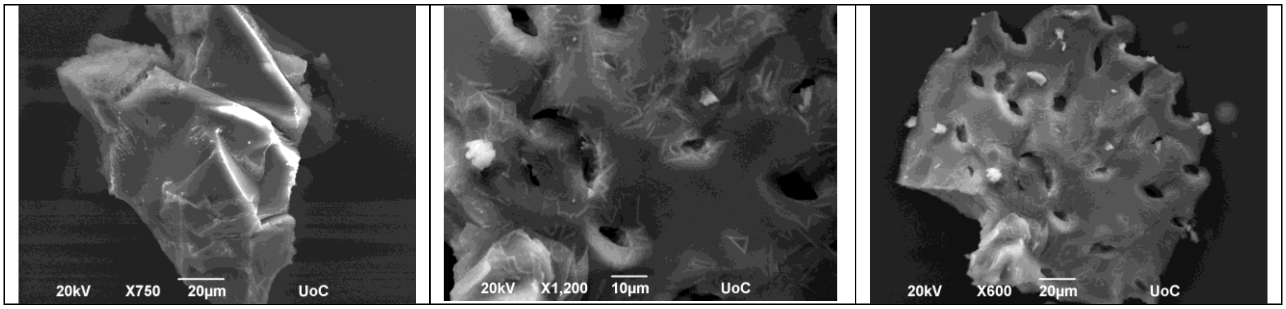

Figure S-2. SEM images (surface and cross-sections) of silica gels prepared in the absence of BP drug.

Freshly “drug-loaded” gels (with ETID bisphosphonate, <u>before</u> release)

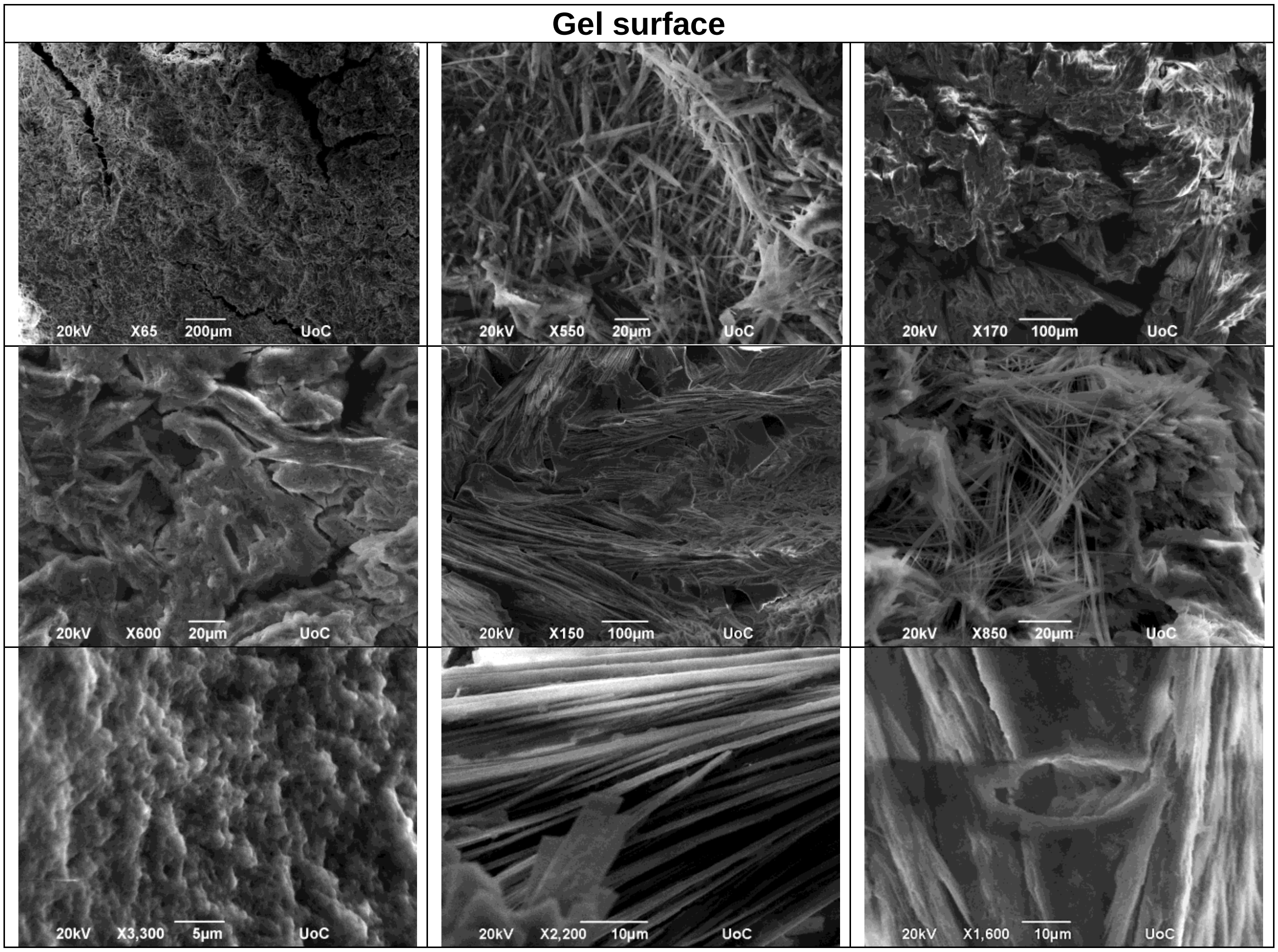

Figure S-3. SEM images of freshly “drug-loaded” gels (with ETID bisphosphonate).

SEM images of “ETID-loaded” gels after 48 hour release.

Gel surface

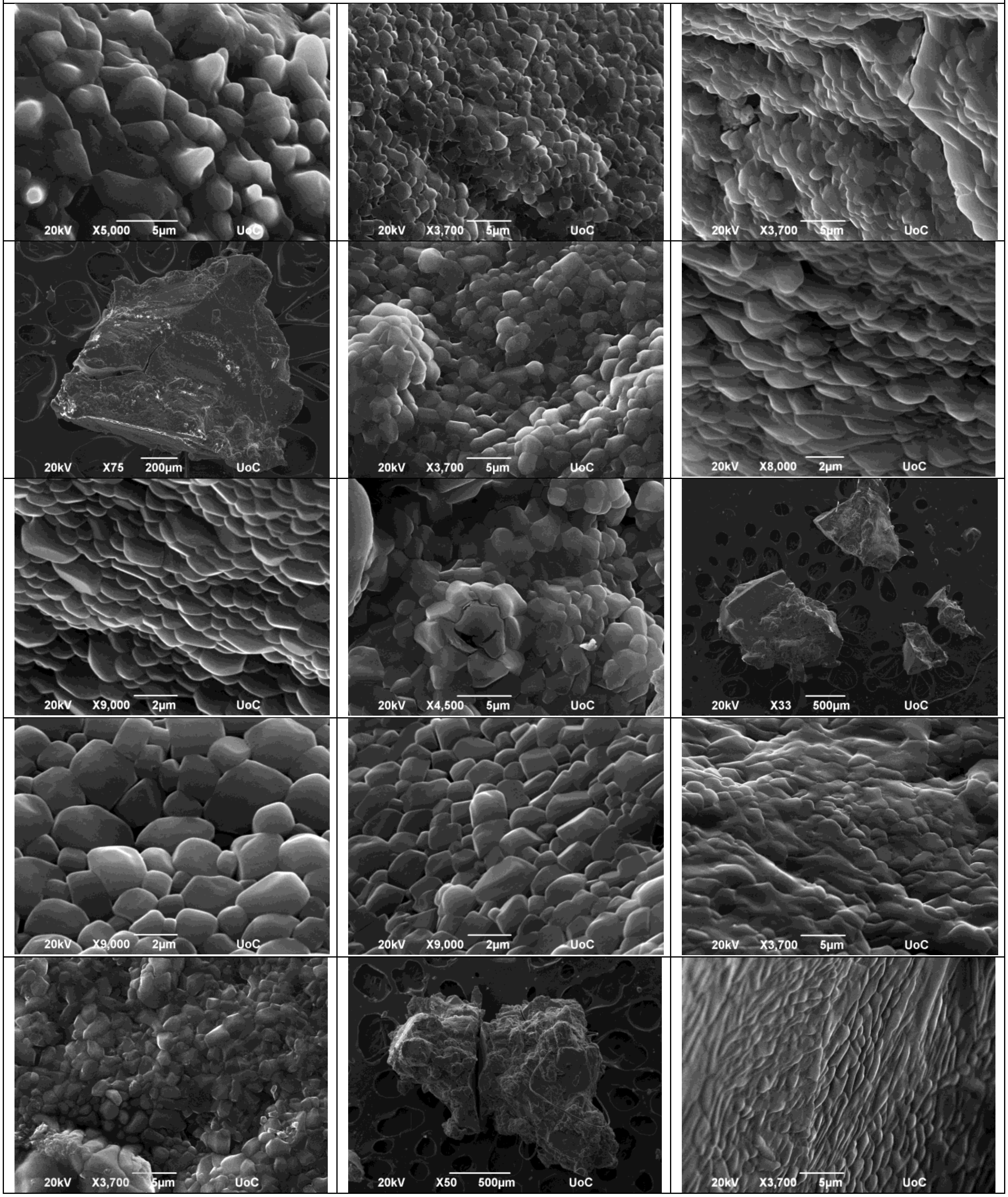

Figure S-4. SEM images of “ETID-loaded” gels after 48 hour release.

EDS characterization

EDS freshly "drug-loaded" gels (with ETID bisphosphonate, before release)

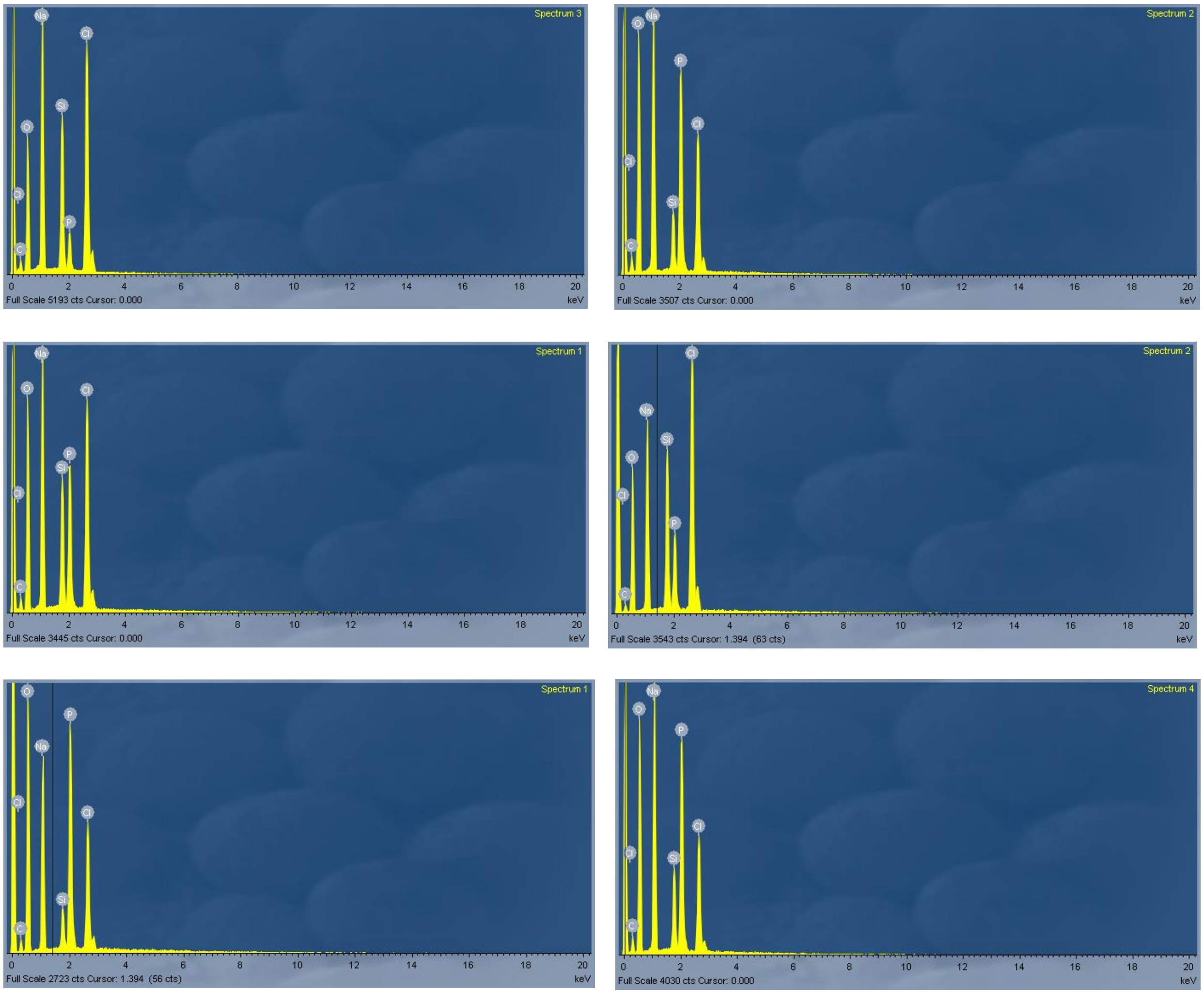

Figure S-5. EDS of freshly-prepared ETID-loaded gels.

EDS of “ETID-loaded” gels after 48-hour release

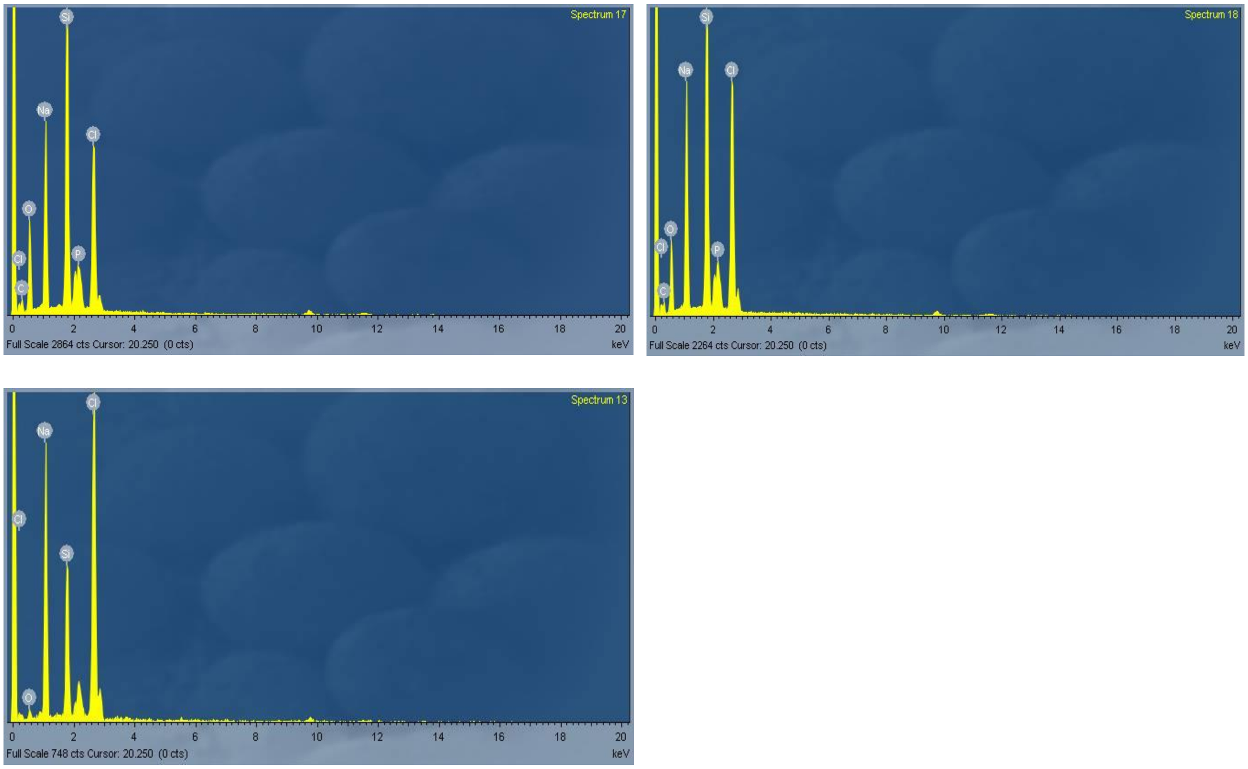

Figure S-6. EDS of ETID-loaded gels **after** 48-hour release.

Rheology of gels

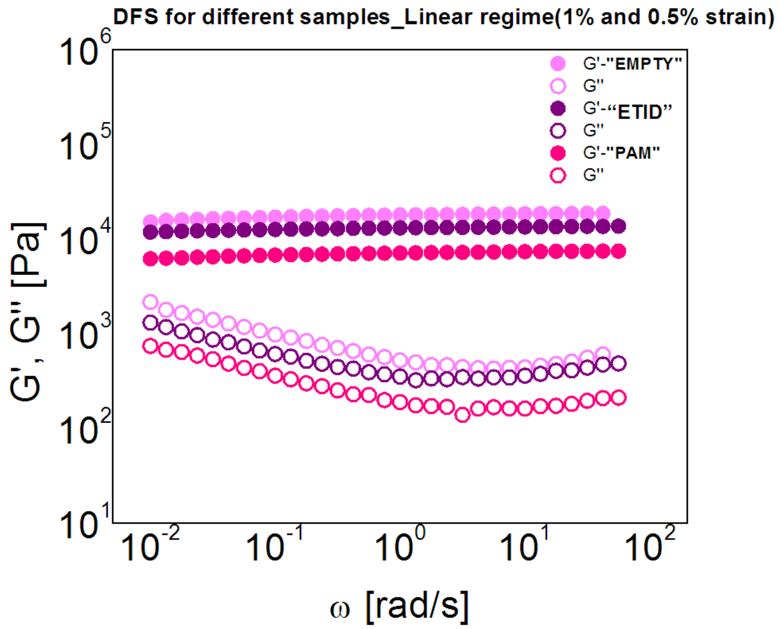

Figure S-7. Comparison of the linear viscoelastic spectra for the three hydrogel samples, "empty", ETID-loaded" and "PAM-loaded".

BET characterization

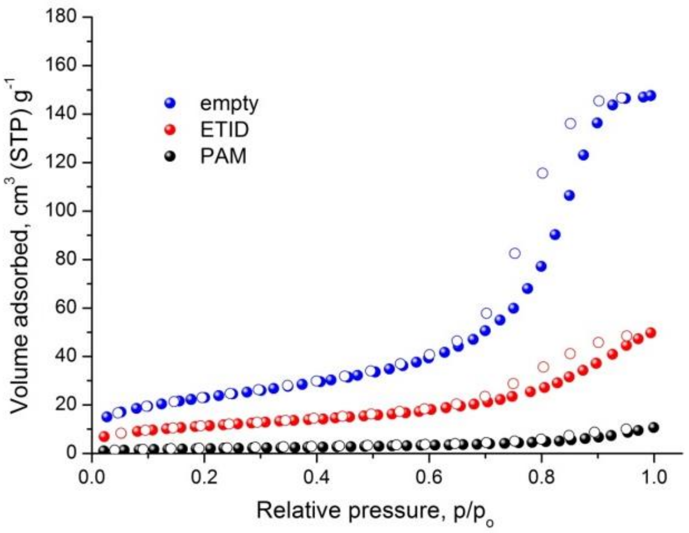

Figure S-8. Nitrogen sorption isotherms for the indicated solids recorded at 77 K.

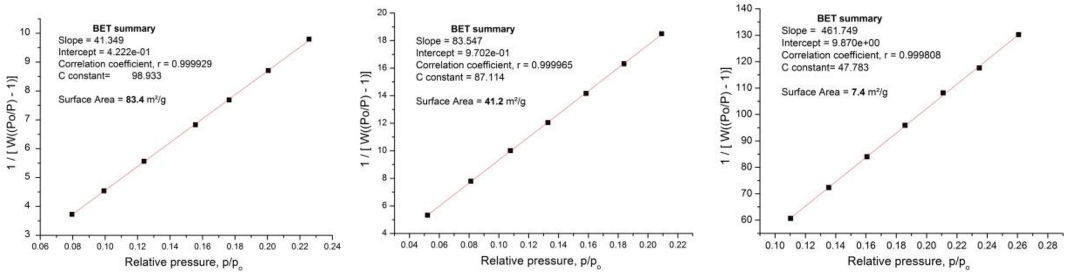

"empty" gel
(SSA = 83.4 m^2/g)

ETID-loaded gel
(SSA = 41.2 m^2/g)

PAM-loaded gel
(SSA = 7.4 m^2/g)

Figure S-9. BET plots for the three "dried" gels calculated from the corresponding N_2 adsorption isotherms.

FT-IR spectra

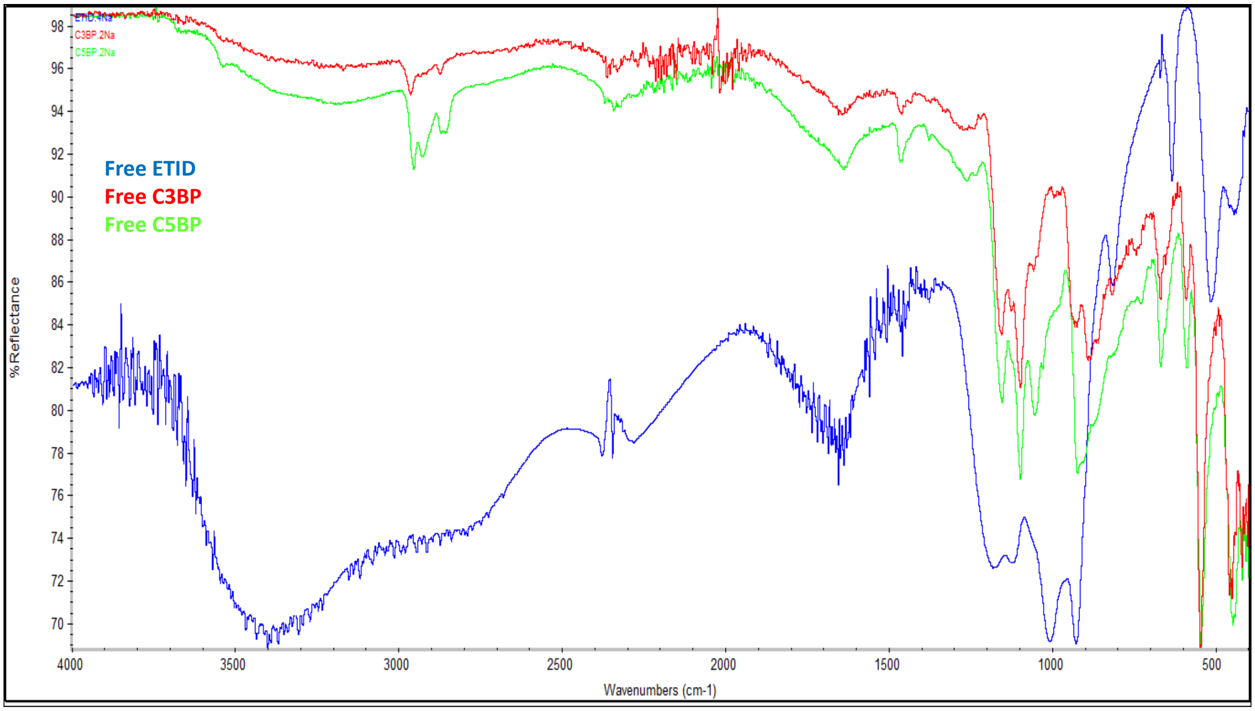

Figure S-10. FT-IR spectra of the "free" BP drugs ETID, C3BP, and C5BP (spectra taken from samples in solid form).

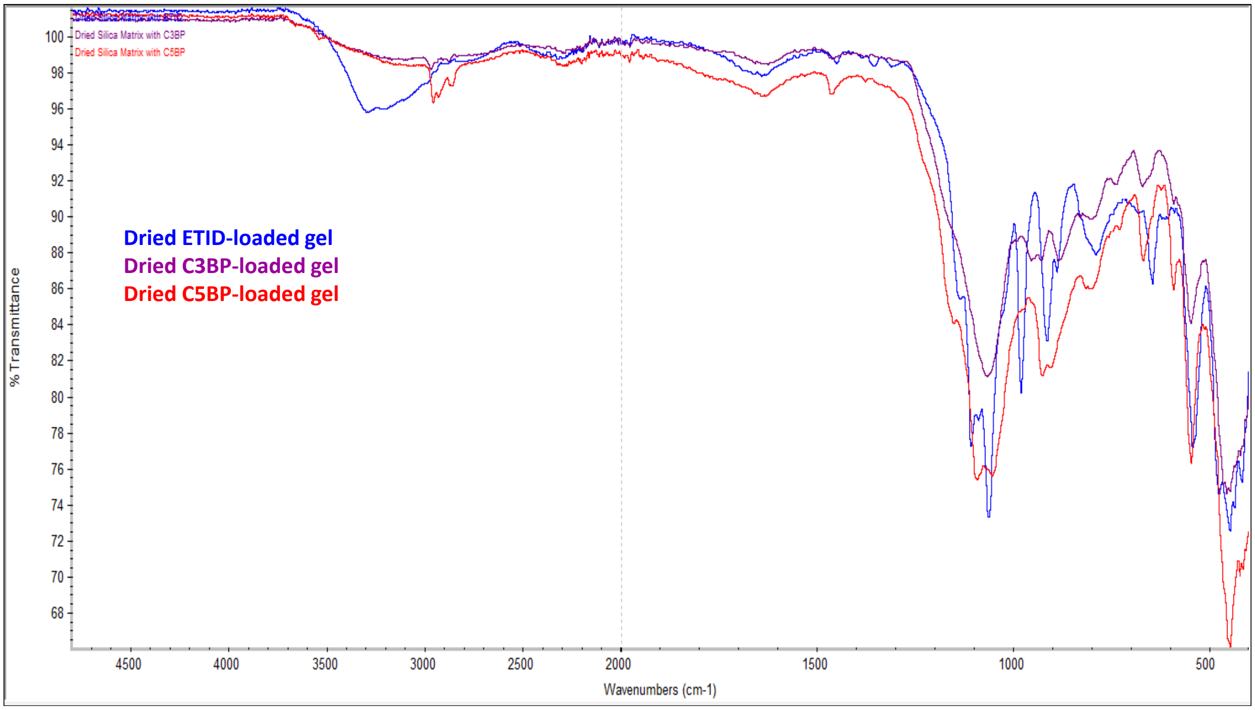

Figure S-11. FT-IR spectra of dried gels: ETID-loaded, C3BP-loaded and C5BP-loaded (spectra taken from samples in solid form).

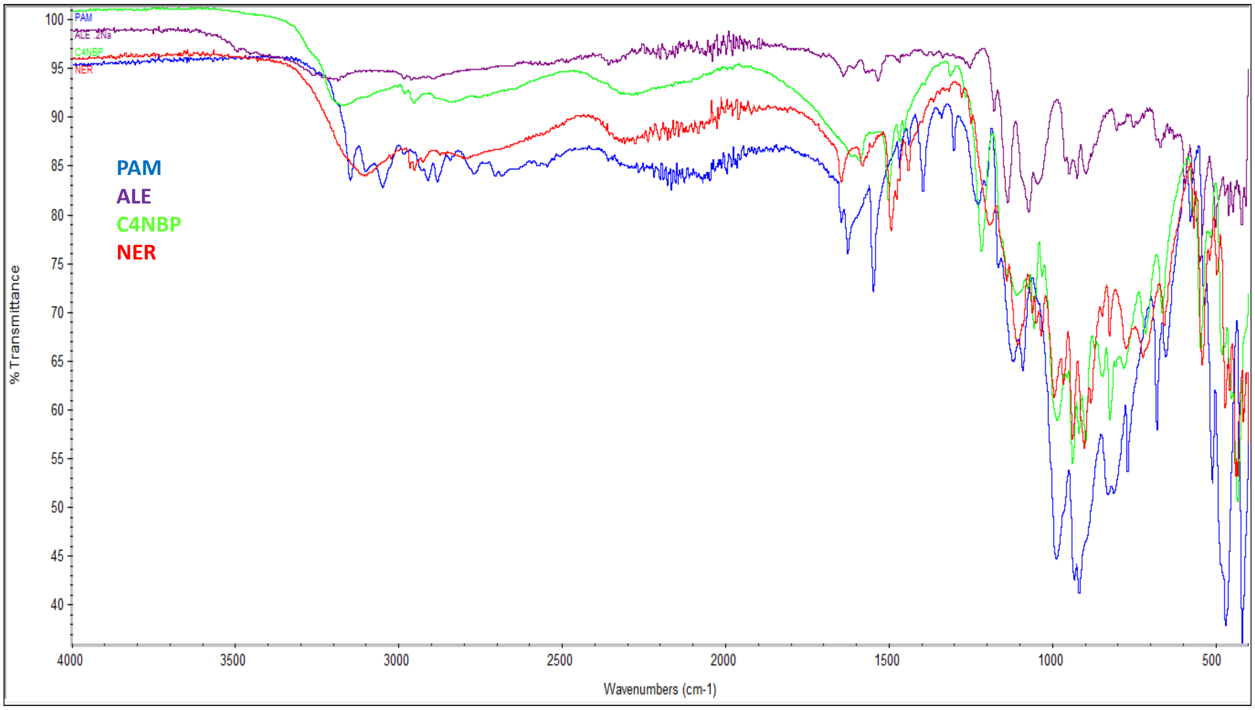

Figure S-12. FT-IR spectra of the “free” BP drugs PAM, ALE, C4NBP, and NER (spectra taken from samples in solid form).

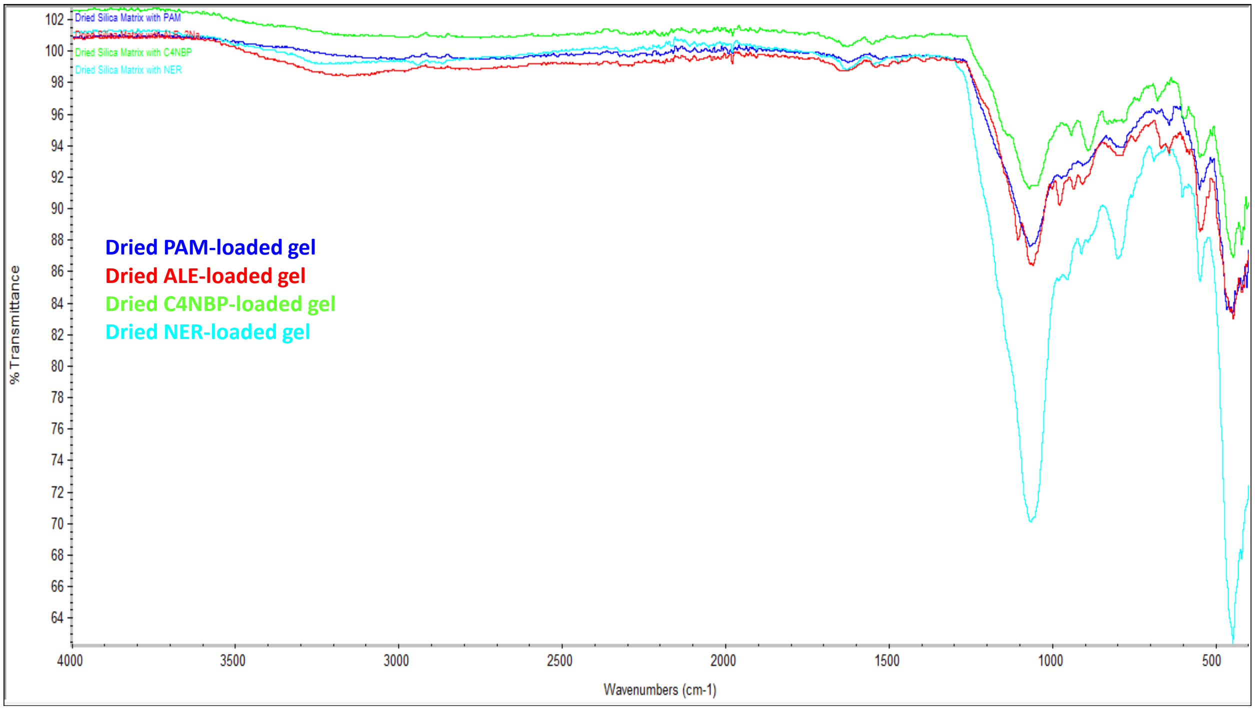

Figure S-13. FT-IR spectra of FT-IR spectra of dried gels: PAM-loaded, ALE-loaded, C4NBP-loaded, and NER-loaded (spectra taken from samples in solid form).

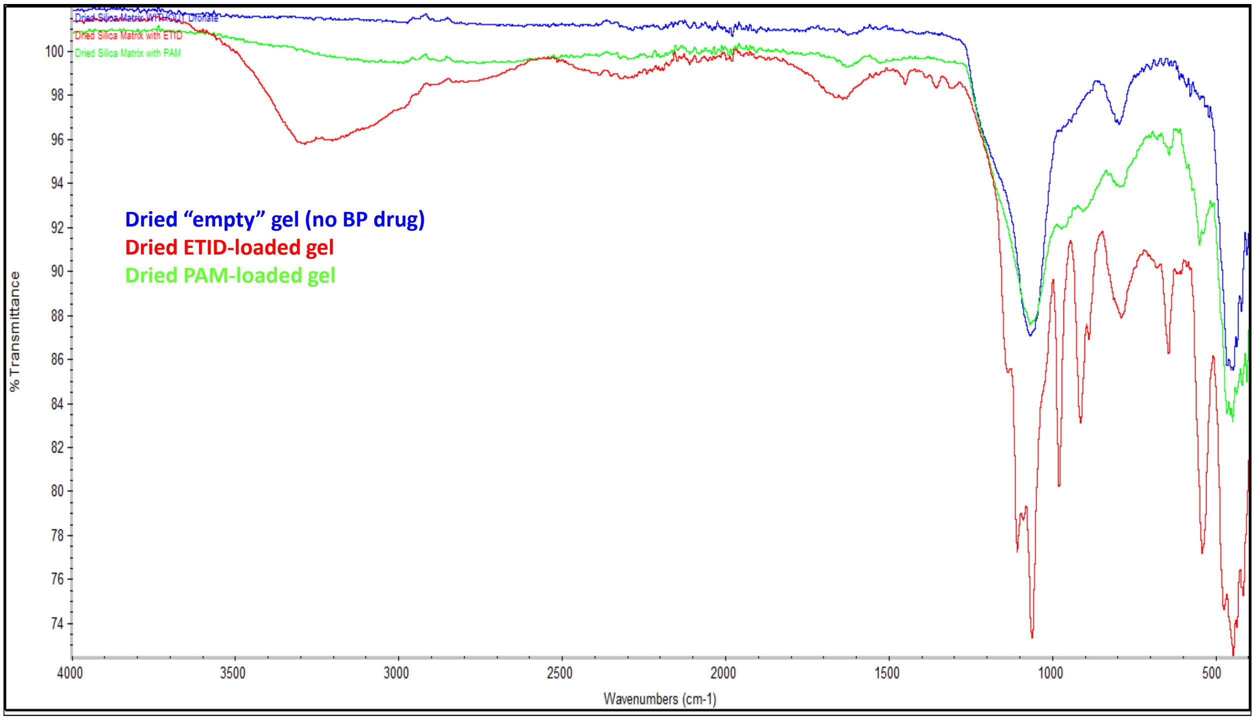

Figure S-14. FT-IR spectra of dried gels: “empty”, ETID-loaded, and PAM-loaded (spectra taken from samples in solid form).

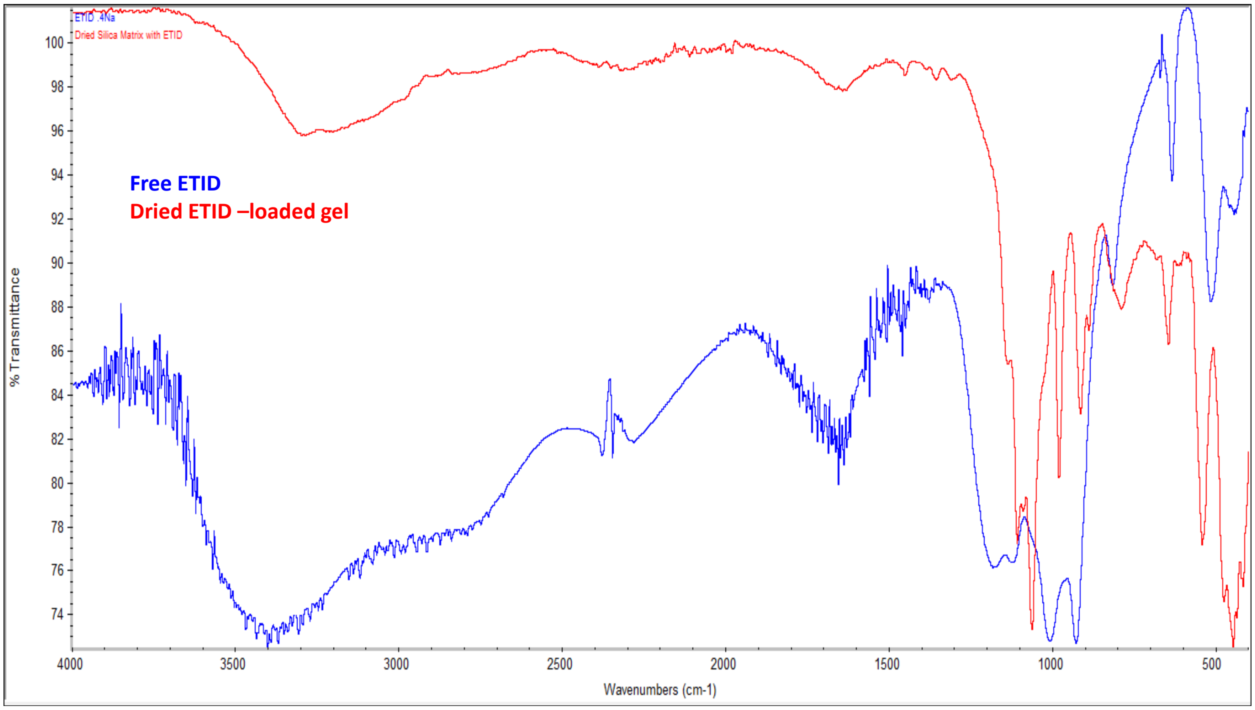

Figure S-15. Comparative FT-IR spectra: Free ETID and ETID-loaded gel (spectra taken from samples in solid form).

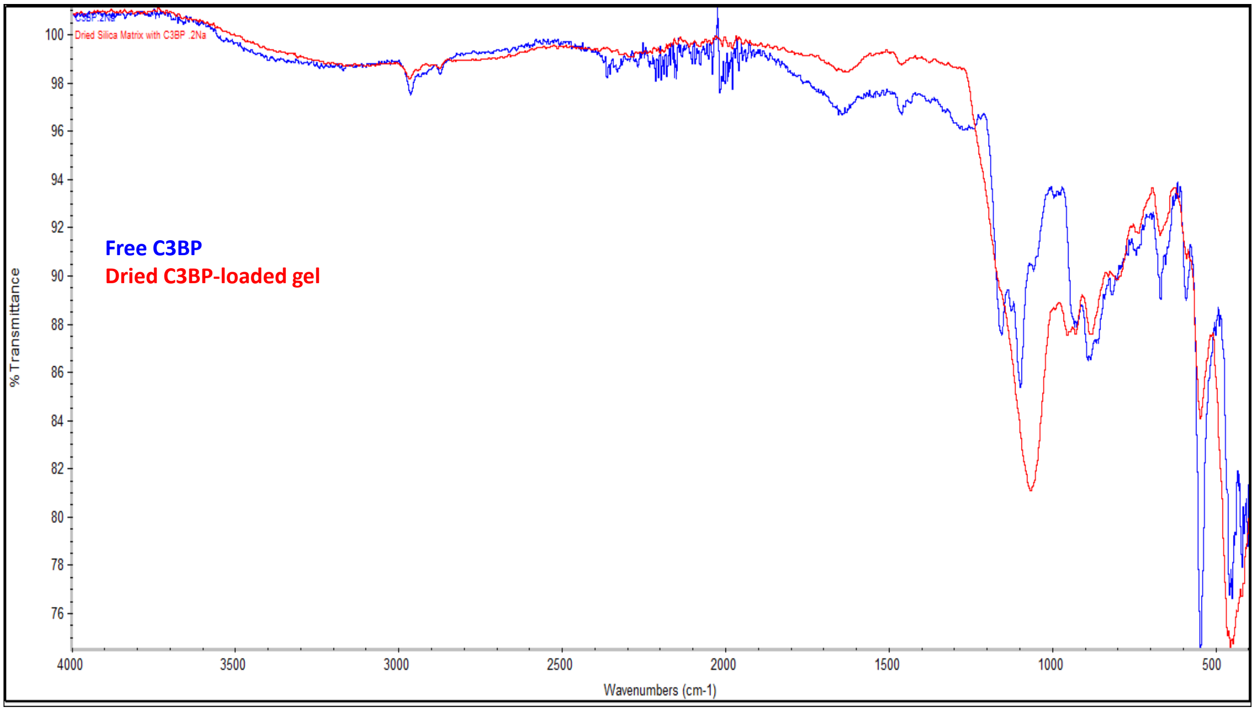

Figure S-16. Comparative FT-IR spectra: Free C3BP and C3BP-loaded gel (spectra taken from samples in solid form).

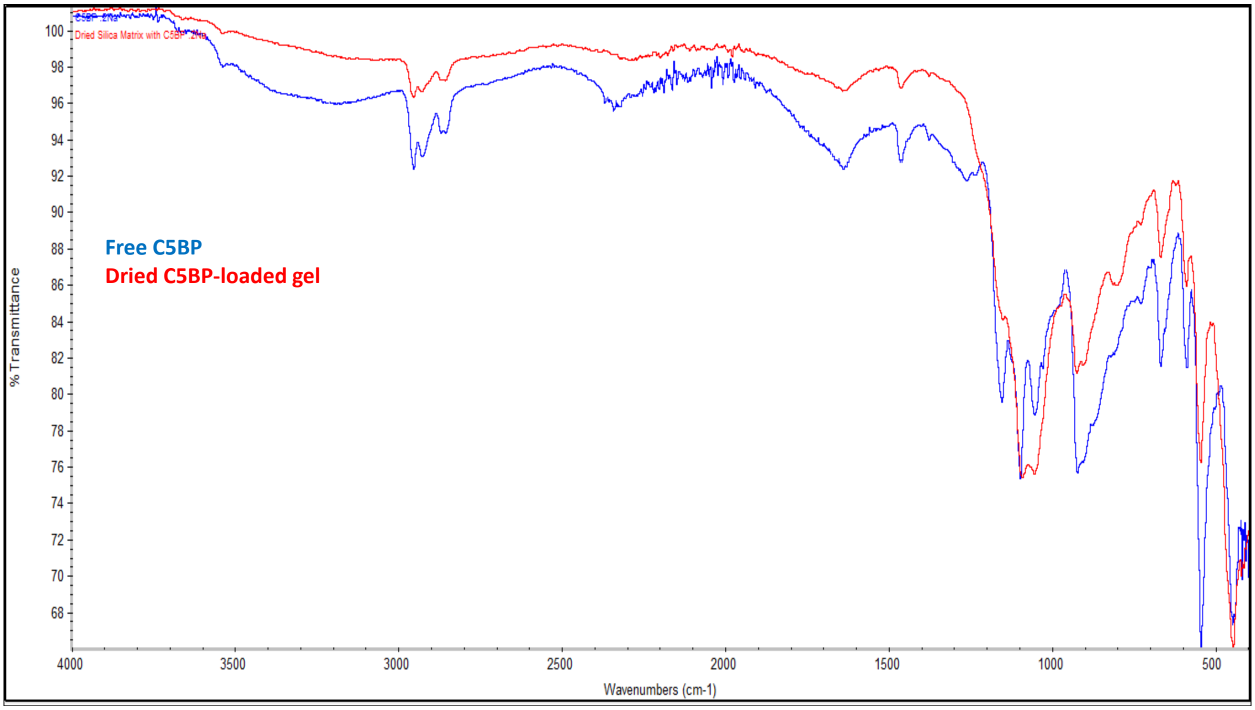

Figure S-17. Comparative FT-IR spectra: Free C5BP and C5BP-loaded gel (spectra taken from samples in solid form).

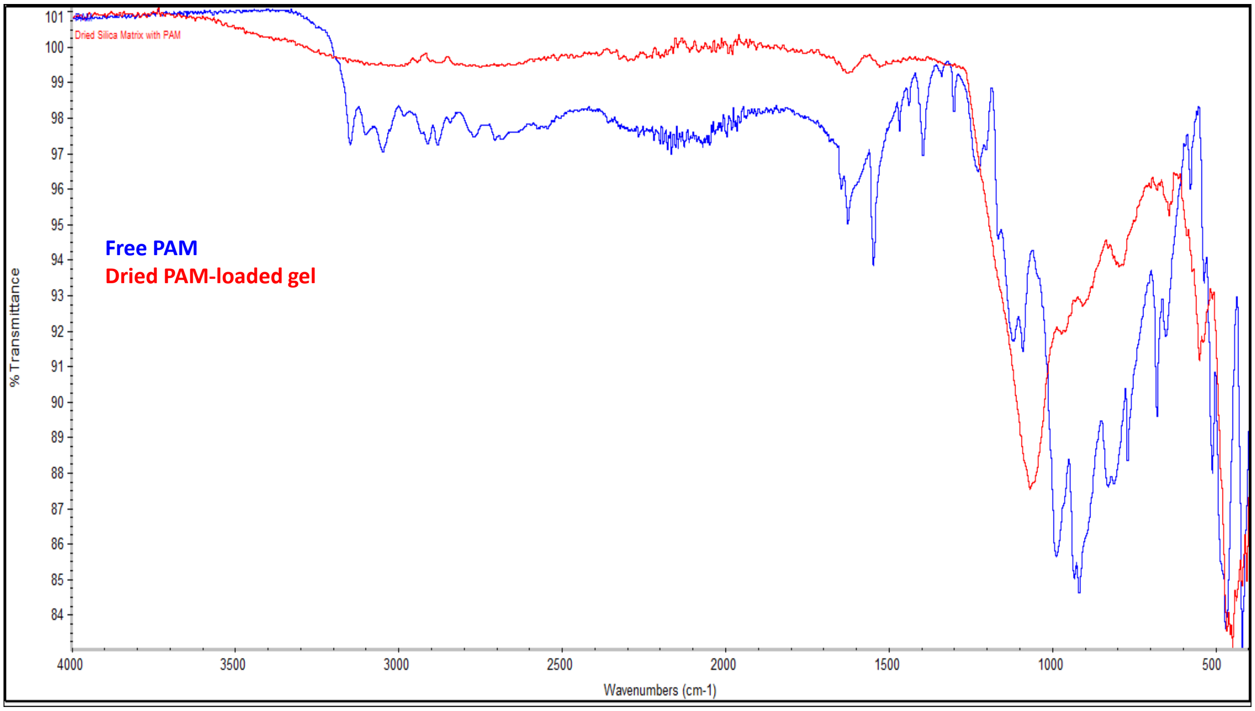

Figure S-18. Comparative FT-IR spectra: Free PAM and PAM-loaded gel (spectra taken from samples in solid form).

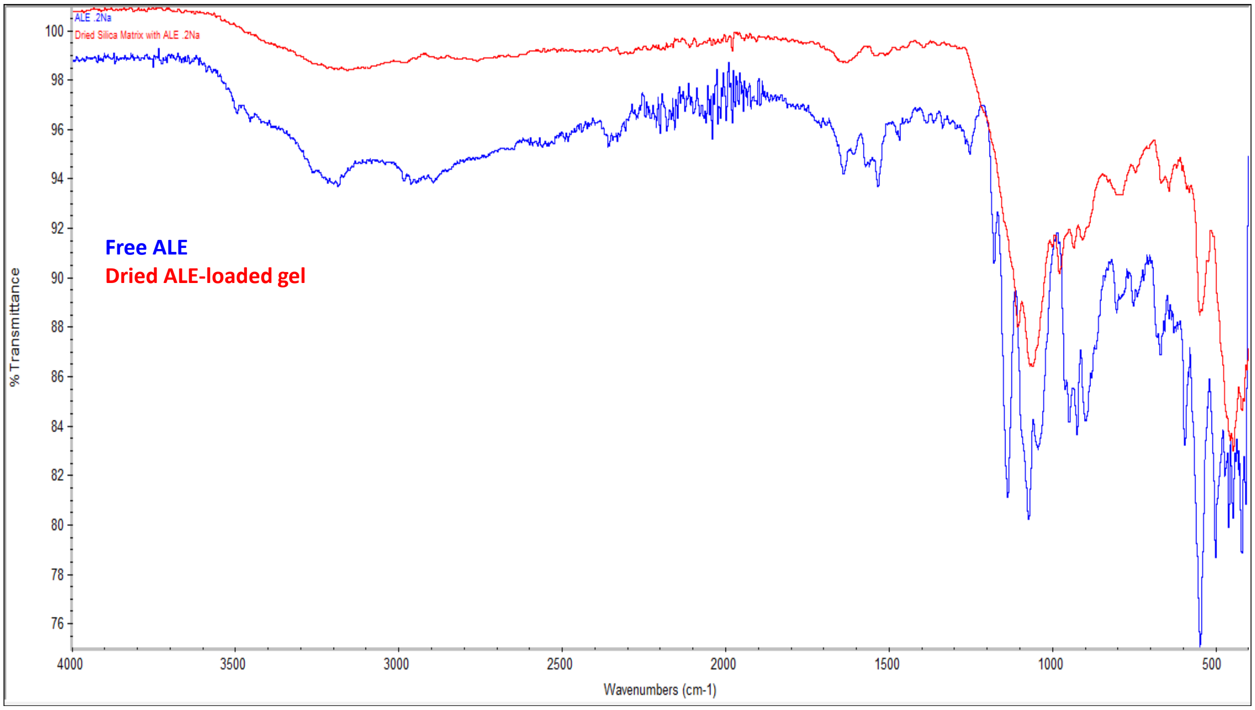

Figure S-19. Comparative FT-IR spectra: Free ALE and ALE-loaded gel (spectra taken from samples in solid form).

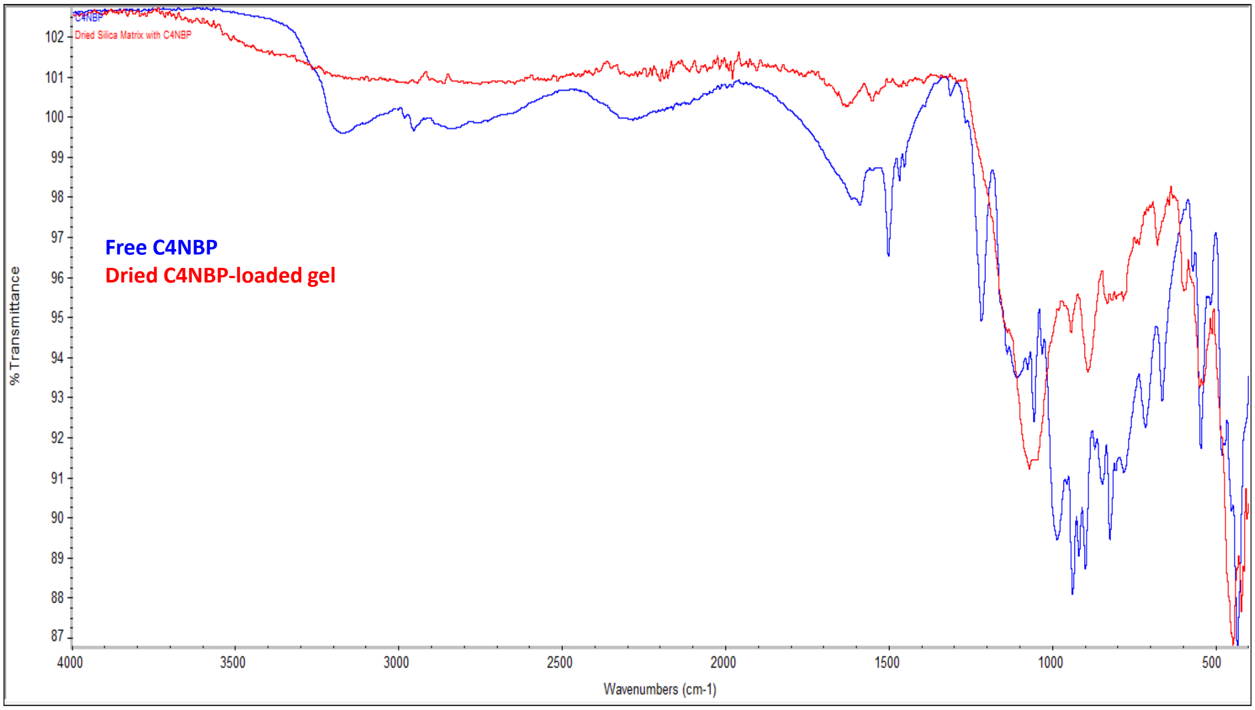

Figure S-20. Comparative FT-IR spectra: Free C4NBP and C4NBP-loaded gel (spectra taken from samples in solid form).

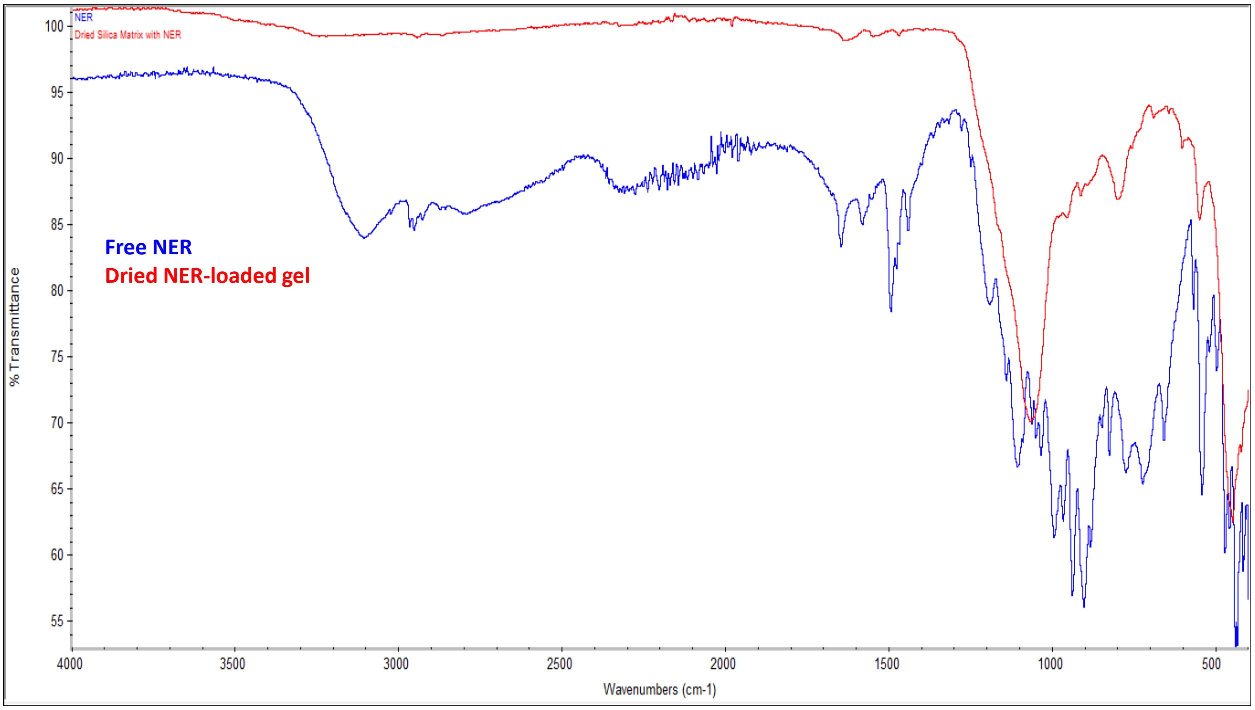

Figure S-21. Comparative FT-IR spectra: Free NER and NER-loaded gel (spectra taken from samples in solid form).

5. Miscellaneous controlled release comparative data

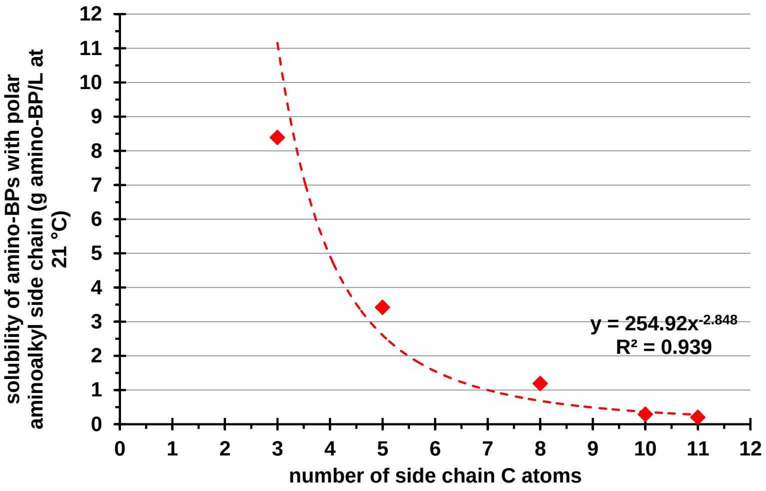

Figure S-22. Correlation between the number of carbon atoms (length of aminoalkyl side chain) on amino-BPs and water solubility. Data taken from the SI of the paper A.-L. Alanne, H. Hyvönen, M. Lahtinen, M. Ylisirniö, P. Turhanen, E. Kolehmainen, S. Peräniemi, J. Vepsäläinen, *Molecules*, **2012**, *17*, 10928-10945.

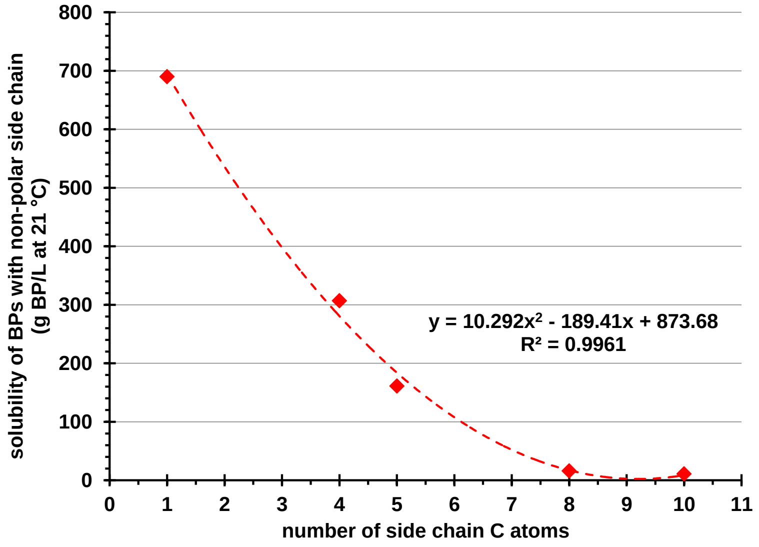

Figure S-23. Correlation between the number of carbon atoms (length of aminoalkyl side chain) on amino-BPs and water solubility. Data taken from the SI of the paper A.-L. Alanne, M. Tuikka, K. Tõnsuaadu, M. Ylisirniö, L. Hämäläinen, P. Turhanen, J. Vepsäläinen, S. Peräniemi, *RSC-Adv.* **2013**, *3*, 14132–14138.

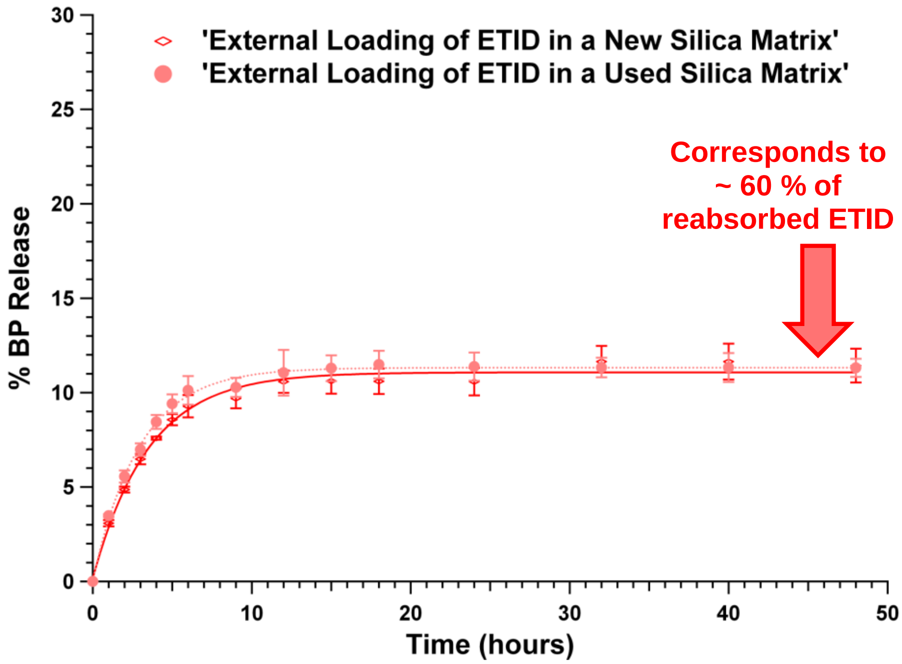

Figure S-24. Release profiles of re-absorbed ETID from a freshly-prepared gel and from a used (emptied) gel.

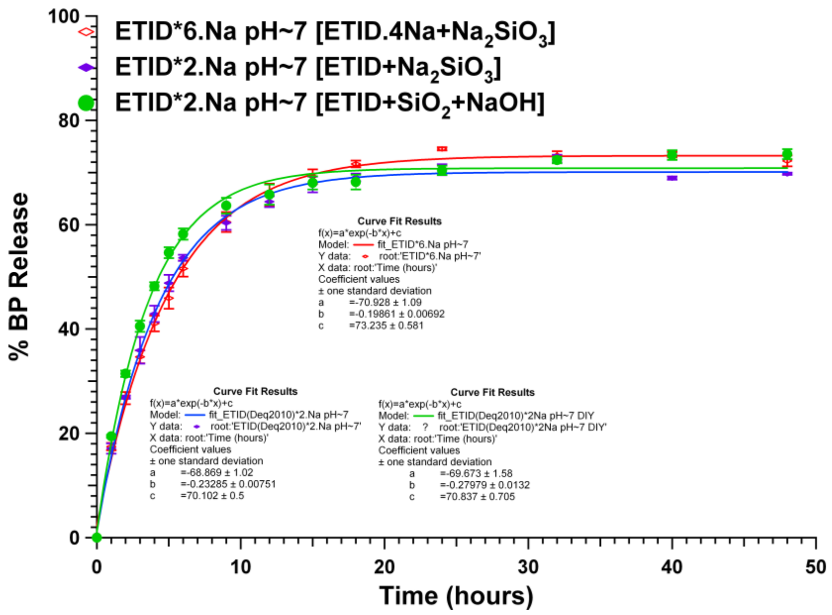

Figure S-25. Effect of number of Na^{+} ions per ETID molecule present in the gel, on ETID release.

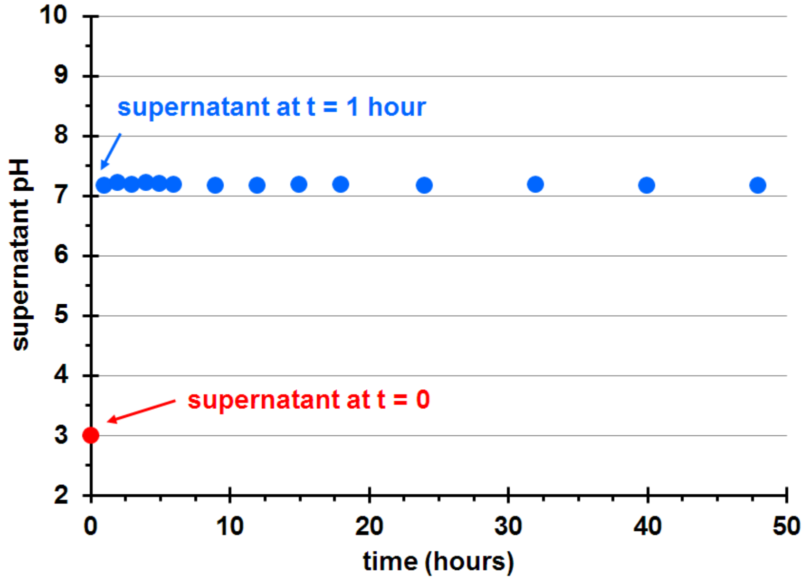

Figure S-26. Evolution of pH in the aqueous supernatant (above the gel mass).

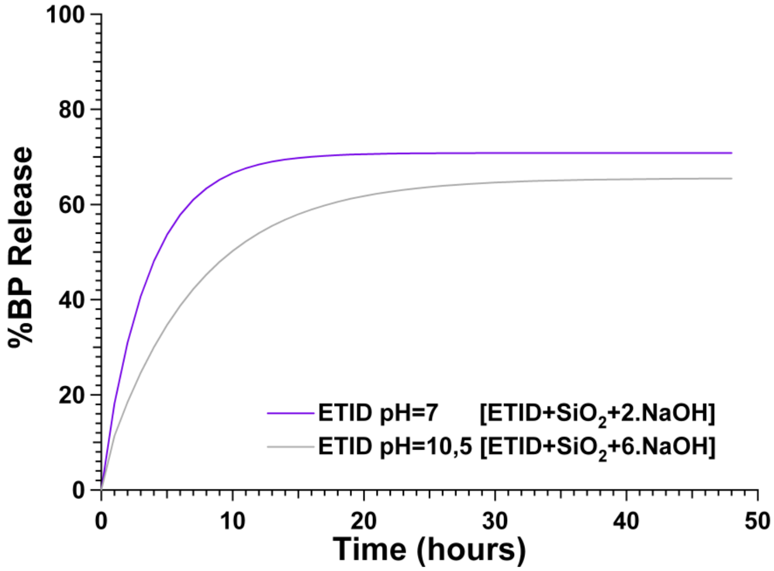

Figure S-27. Effect of supernatant pH on ETID release.

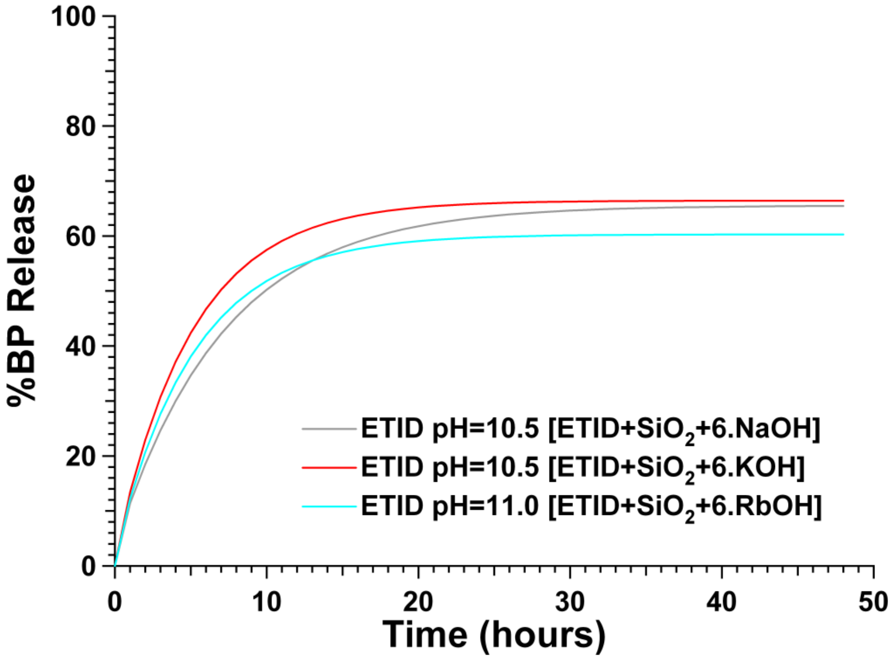

Figure S-28. Effect of the nature of alkali cation on ETID release. The K- and Rb- gels were fabricated at pH 10.5 – 11.0, because it was not possible to prepare them at pH = 7.0.

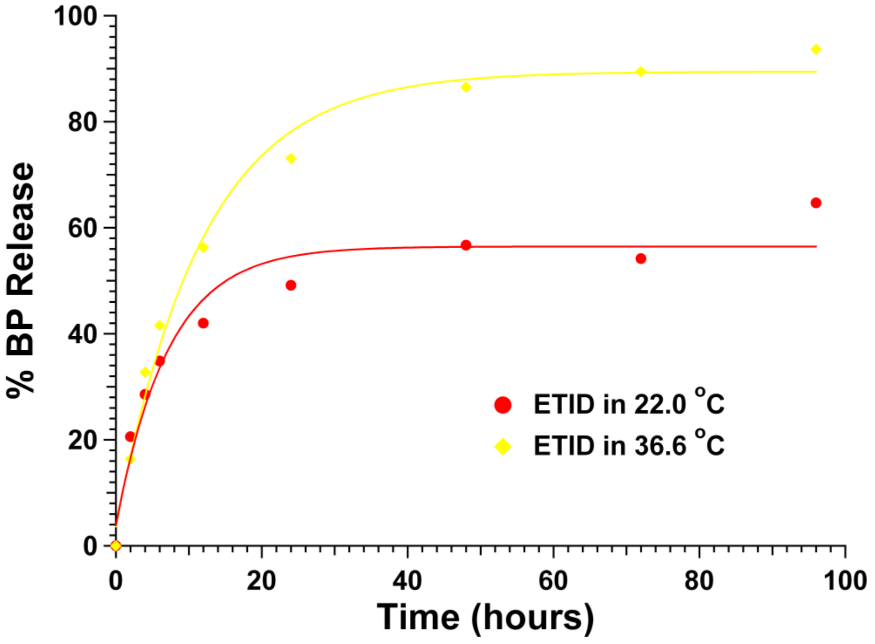

Figure S-29. Effect of temperature on the release of ETID.

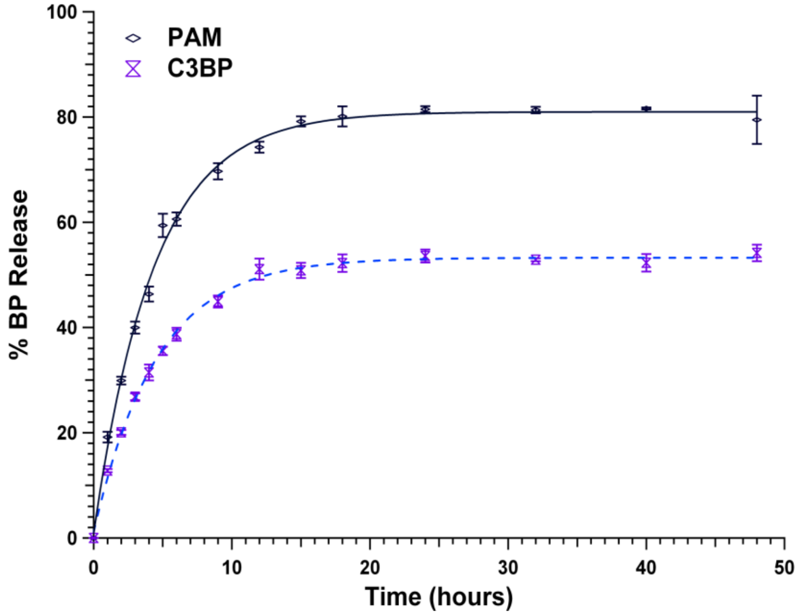

Figure S-30. Comparison of release profiles of C3BP (a 3-atom, non-polar alkyl side-chain) and PAM (a 3-atom, amine-containing polar side-chain).

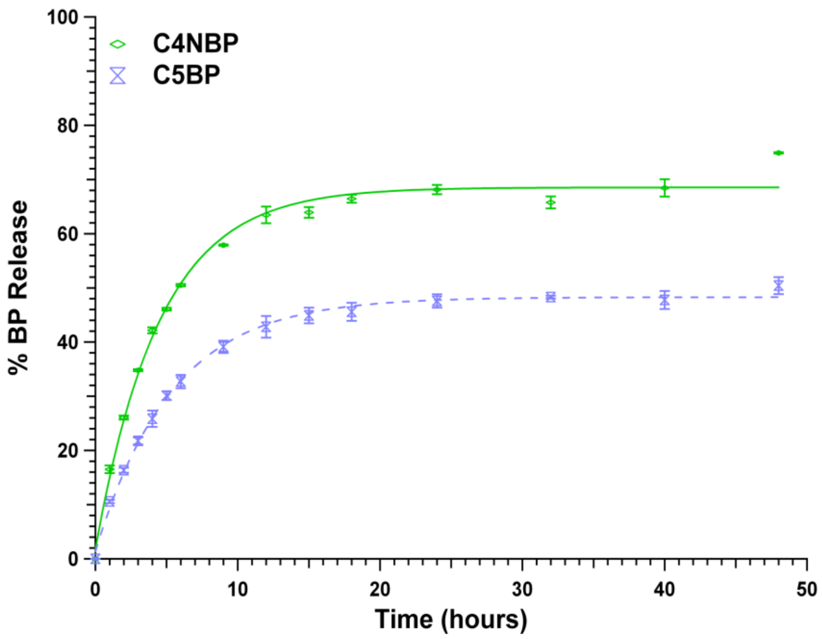

Figure S-31. Comparison of release profiles of C5BP (a 5-atom, non-polar alkyl side-chain) and C4NBP (a 5-atom, amine-containing polar side-chain).

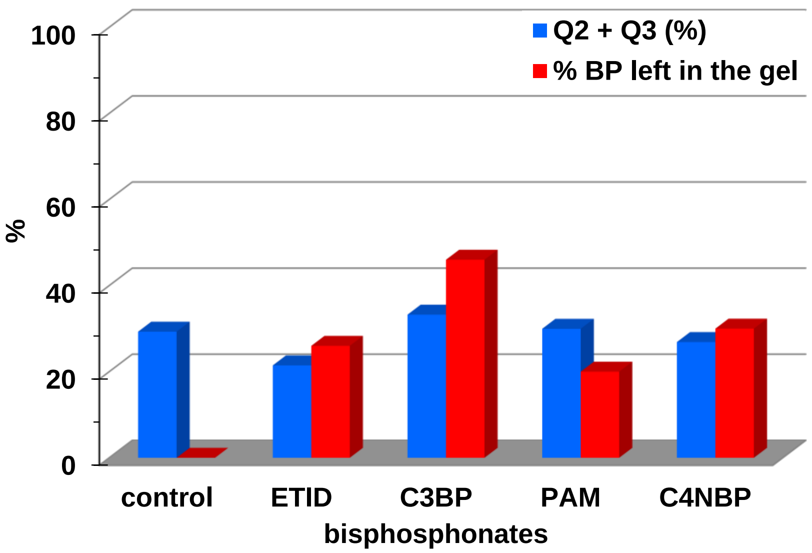

Figure S-32. Correlation between percent of Q2 + Q3 in various gels and % BP left in the gel after a 48-hour release.

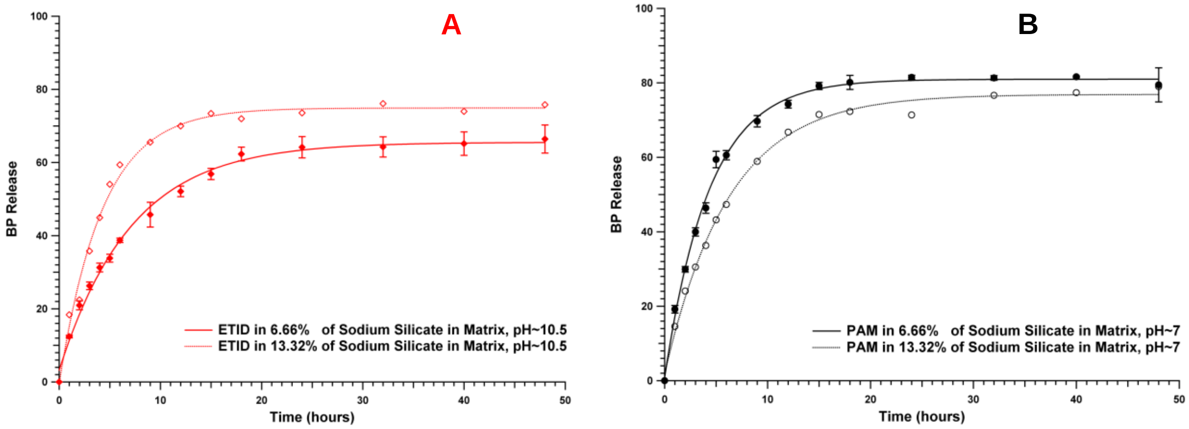

Figure S-33. Comparison of the BP drug release curves for "normal" (6.66 % sodium silicate, solid curves) and "dense" (13.32 % sodium silicate, dashed curves) ETID- and PAM-loaded gels.

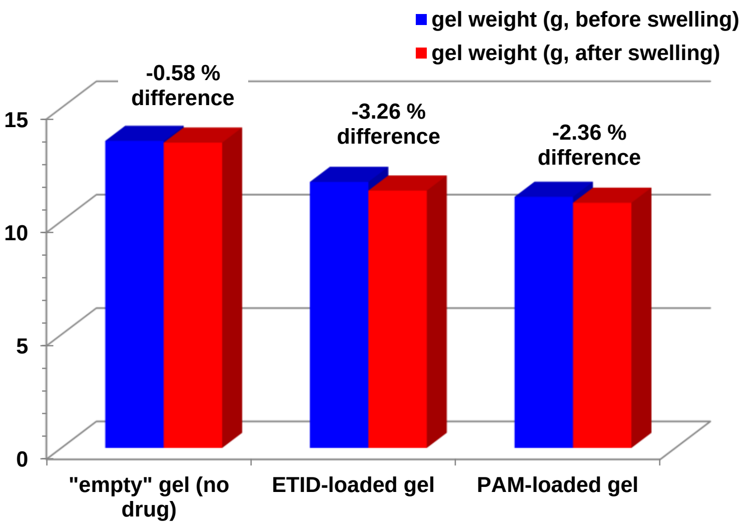

Figure S-34. Water absorption from freshly formed gels. Essentially, there is no swelling.

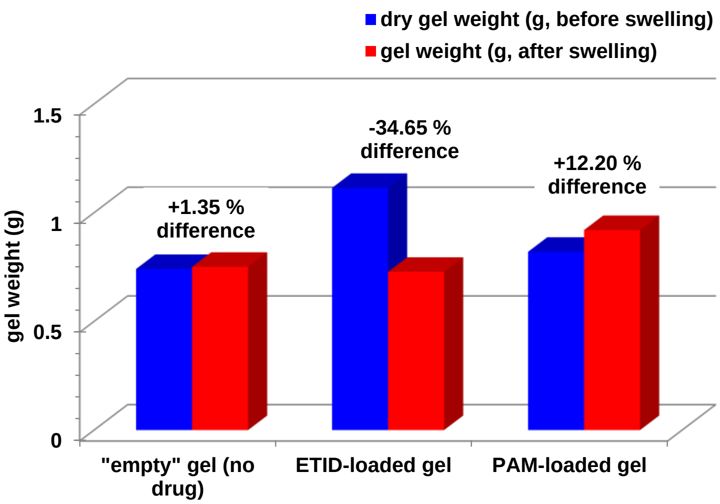

Figure S-35. Water absorption from dried gels.

6. Mathematical data treatment

All aliquot samples withdrawn from the supernatant were measured using ^{1}H, ^{13}C, and ^{31}P NMR spectroscopy. The quantification of the released BP was based on integration of peaks in the ^{1}H spectrum and was made possible by using deuterium oxide 99.9 atom % D as solvent, that contains 0.05 wt. % 3-(trimethylsilyl)propionic-*2,2,3,3-d*$_4$ acid sodium salt, TSP, as standard. Each release experiment was repeated 4 times in order to achieve maximum reproducibility and satisfactory statistics. Each release experiment consisting of 15 samplings/measurements, and repeated 4 times, was treated with the IGOR Pro 6.05 software.

The last step in the mathematical treatment of the results was the creation of a universal curve in the form $f(t) = a * e^{-b*t} + c$ that depicts the average value of the diffusion of the phosphonate from the silica hydrogel, including standard deviation. Factor "a" of the equation is the “frequency factor” and it is related to the entropy difference between the gel and the liguid phase. Factor “b” is the exponential parameter that describes the energy statistical distribution of the molecules through desorption. Constant "c" describes all the remaining interactions between the different phases (diffusion inside the gel, diffusion in the liquid, adsorption etc). The variable “t” is the time.

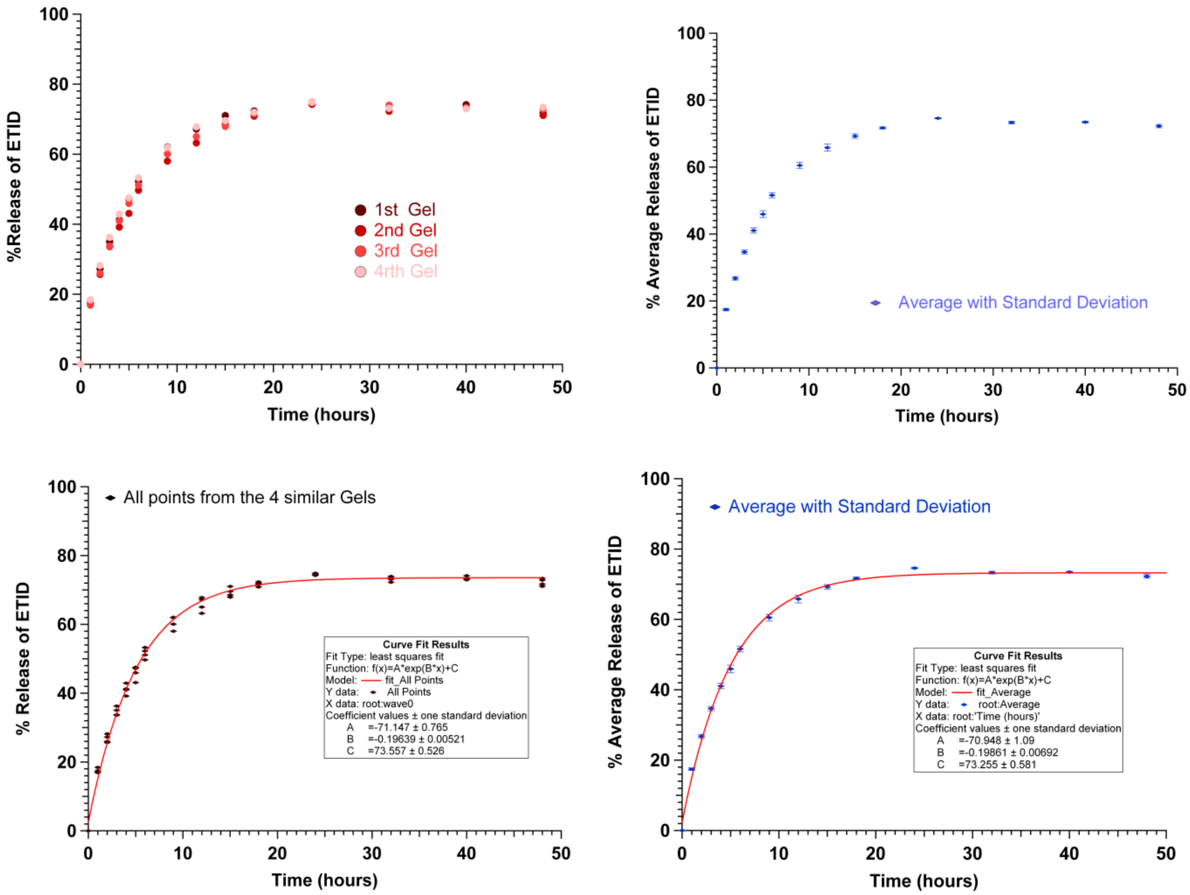

Figure S-36. The least square theory applied by the IGOR software on all data points (graphs to the left) and mean values (graphs to the right). In both cases the results are almost identical.

7. **Gel NMR (^{31}P MAS and ^{1}H-^{31}P CP MAS) spectra**

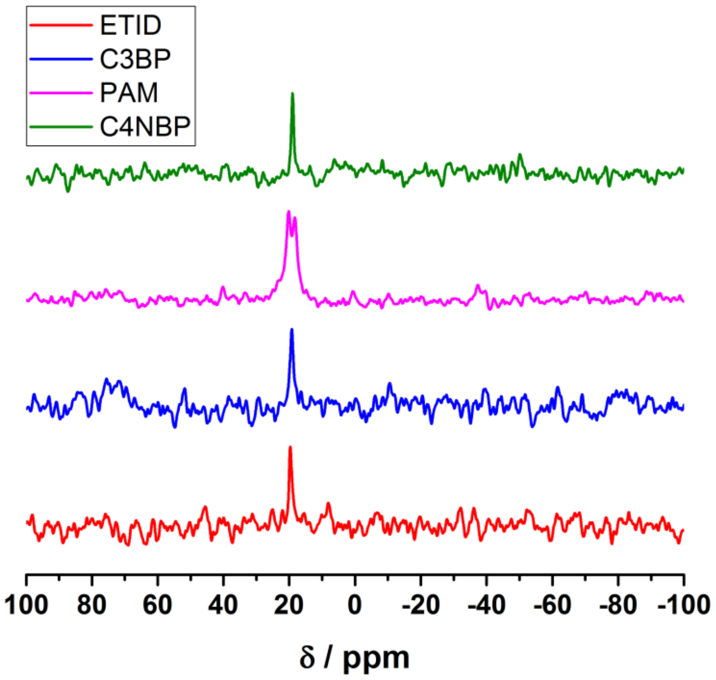

Figure S-37. Directly excited ^{31}P MAS NMR spectra of selected pure BPs after incorporation in the silica gel.

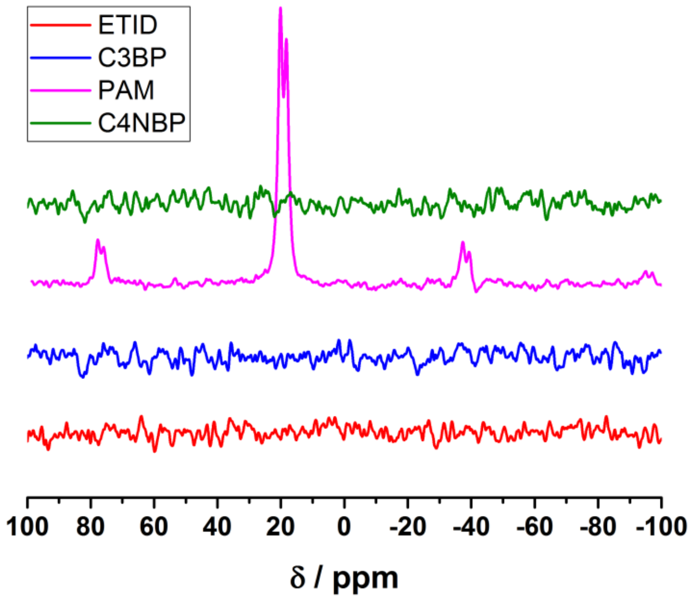

Figure S-38. ^{1}H-^{31}P CP MAS NMR spectra of selected pure BPs after incorporation in the silica gel. These spectra were recorded using cross polarization, in which the corresponding transfer of magnetization is only efficient for rigid/solid samples. Hence, the presence of peaks in the PAM case (in contrast to the other BPs) means that it most likely has crystallized in the rotor during the ssNMR measurements.
